# Biomimetic reconstruction of the hematopoietic stem cell niche for *in vitro* amplification of human hematopoietic stem cells

**DOI:** 10.1371/journal.pone.0234638

**Published:** 2020-06-22

**Authors:** L. Marx-Blümel, C. Marx, F. Weise, J. Frey, B. Perner, G. Schlingloff, N. Lindig, J. Hampl, J. Sonnemann, D. Brauer, A. Voigt, S. Singh, B. Beck, Ute-Maria Jäger, Z. Q. Wang, J. F. Beck, A. Schober

**Affiliations:** 1 Department of Paediatric Haematology and Oncology, Children’s Clinic, Jena University Hospital, Jena, Germany; 2 Research Center Lobeda, Jena University Hospital, Jena, Germany; 3 Leibniz Institute on Aging—Fritz Lipmann Institute (FLI), Jena, Germany; 4 Institute for Micro and Nanotechnologies MacroNano^®^, Nano-Biosystem Technology, Ilmenau University of Technology, Ilmenau, Germany; 5 Faculty of Biological Sciences, Friedrich-Schiller-University of Jena, Jena, Germany; Università degli Studi della Campania, ITALY

## Abstract

Hematopoietic stem cell transplantation is successfully applied since the late 1950s; however, its efficacy still needs to be increased. A promising strategy is to transplant high numbers of pluripotent hematopoietic stem cells (HSCs). Therefore, an improved *ex vivo* culture system that supports proliferation and maintains HSC pluripotency would override possible limitations in cell numbers gained from donors. To model the natural HSC niche *in vitro*, we optimized the HSC medium composition with a panel of cytokines and valproic acid and used an artificial 3D bone marrow-like scaffold made of polydimethylsiloxane (PDMS). This 3D scaffold offered a suitable platform to amplify human HSCs *in vitro* and, simultaneously, to support their viability, multipotency and ability for self-renewal. Silicon oxide-covering of PDMS structures further improved amplification of CD34+ cells, although the conservation of naïve HSCs was better on non-covered 3D PDMS. Finally, we found that HSC cultivated on non-covered 3D PDMS generated most pluripotent colonies within colony forming unit assays. In conclusion, by combining biological and biotechnological approaches, we optimized *in vitro* HSCs culture conditions, resulting in improved amplification, multipotency maintenance and vitality of HSCs.

## Introduction

Human hematopoietic stem cells (HSCs) are able to produce all types of blood cell lineages and are defined by two key properties: the ability of self-renewal and their potential of lineage specific differentiation [1, 2]. Therefore, HSCs are an appealing source for stem cell-based therapies such as hematopoietic stem cell transplantation (HSCT) [3].

Since its introduction in the late 1950s, the procedure of HSCT has dramatically improved. It has become a potentially life-saving treatment option for patients with otherwise incurable diseases like chemoresistant high-risk malignancies and a broad variety of non-malignant congenital and acquired disorders [4]. Efforts to further progress HSCT are ongoing. A very promising approach may lie in the transplantation of high numbers of pluripotent stem cells to minimize the time span between transplantation and immunological reconstitution [5]. This can be achieved by an *ex vivo* expansion of cultured stem cells before transplantation. However, the attempts to expand undifferentiated stem cells *in vitro* have been of limited success so far [3]. A particular challenge is that HSCs rapidly differentiate *in vitro* to become committed progenitor cells, which lost their ability for self-renewal.

Several approaches have already been done to improve HSC culture conditions. Advanced cultivation protocols include the use of hematopoietic cytokines, developmental regulators and/or chemical compounds to maintain HSC pluripotency. However, these approaches are not fully sufficient to both support the ability of self-renewal and effective amplification of HSCs *in vitro* [3]. Other approaches rely on the use of 3D cultivation chambers that mimic the natural bone marrow (BM) niche, based on the hypothesis that the culture environment may play a critical role in the expansion of HSCs *in vitro* [6–9]. Various natural biomaterials and synthetic scaffolds have been described to create close-to-nature microenvironments for an improved control of stem cell response *in vitro* [9–11].

Therefore, we optimized the HSC culture medium to improve cell expansion and designed a 3D structure made of polydimethylsiloxane (PDMS) based on a human long bone cross section to mimic the natural stem cells niche and thus, to maintain larger amounts of undifferentiated HSCs.

## Material and methods

### Cell culture

Cryoconserved pre-isolated human CD34+ HSCs were obtained from mobilized peripheral blood (mPB) of male and female healthy donors from the Cryogenic Storage Facility (Biobank) of the Children’s Hospital Jena. The study has been approved by the Jena University Hospital Ethics Committee (#3595-10/12, #5320-10/17).

Cells were cultured in HSC medium (Stemline^®^ II Hematopoietic Stem Cell Expansion Medium (Sigma Aldrich) supplemented with 100 U/ml penicillin and 100 U/ml streptomycin (Lonza), 100 ng/ml SCF (Peprotech), 100 ng/ml TPO (Peprotech), 100 ng/ml FLT-3 (Peprotech), 50 ng/ml IL-3 (Peprotech)) at 37°C and 5% CO_2_ for an adaption period of 24 h prior to experiments. For experiments, 1x10^4^ cells per well were seeded (day 0) into 24-well polystyrene (PS) cell culture plates (Greiner) in 1 ml HSC medium and cultured for up to 14 days *in vitro* (DIV). Depending on cell densities, medium was replaced or cells were passaged at day 8. Cell numbers were determined 24 h after thawing and after 14 DIV routinely on a Medonic M-series M32 cell counter (Boule Diagnostics). Considering the dilution factor from passaging at day 8, cell expansion after 14 DIV was determined.

To enhance stem cell maintenance, HSC medium was supplemented with 0.5 and 1 mM valproic acid (VPA; Sigma Aldrich) or 0.5 and 1 μM stemreginin-1 (SR-1; Stemmcell) as well as with their combinations.

### Production of PDMS scaffolds

Images of a microtome intersection (10 μm) from the bone marrow (BM) of a human long bone were analyzed by image processing tools from Matlab^®^ and "Mathworks File Exchange". Images were converted to grayscale to create a binary image using a threshold filter (Niblack) and vectorized by CorelDraw^®^. By processing this vector data with AutoCAD^®^, BM structures remained, but the number of sampling points was reduced. The original image (1 mm x 1 mm) was patterned and aligned into a 2D array fitting the diameter of one well of a 24-well plate (1.9 cm^2^) and extracted features were adapted to construct photolithographic masks. By micro structuring with an ICP process, silicon masters were fabricated and utilized as either casting or stamping molds. In this way the construction of positive and negative replicates of the image were done.

Production of the artificial scaffold for HSC cultivation was done by casting and curing a thin silicone layer (1 mm) on top of the silicon BM masters. Due to its excellent optical and molding capabilities, PDMS was chosen [12]. PDMS preparation was done following the manufacturer’s instructions (SYLGARD™ 184 Silicone Elastomer Kit from Dow Inc., Farnell). To avoid air bubbles, freshly mixed PDMS was incubated under vacuum for 30 minutes prior pouring. A molding frame was created to facilitate demolding of the cured PDMS and to achieve highly reproducible results. Underneath this frame, a silicon master (Si-Master) was mounted; 6.3 g of degassed PDMS was poured onto the Si-Master and evacuated again to destroy any trapped air bubbles inside the structures. Afterwards, the mold was heat cured for 15 minutes at 150°C. Heating and cooling steps were performed slowly to avoid any stress inside the material. The described procedure has been patent-registered (Deutsches Patent- und Markenamt: DE 10 2015 122 375 A1).

To achieve a more hydrophilic surface of the PDMS structure, a silicon oxide-based coating called Aquacer^®^ (APC GmbH; SiOn-covering) was used. The Aquacer^®^ coating offers long-term stability and creates a highly hydrophilic surface reducing the contact angle from 107° to less than 10° [13].

### Cell cultivation on PDMS structures

PDMS structures were washed in a descending series of ethanol dilutions (99%, 75%, 50% and 25% in H_2_O) and were placed into 24-well plates (Greiner). Cells were seeded into blank wells (2D PS) for control, SiOn-covered or uncovered 2D and 3D PDMS structures, as well as fibronectin (R&D Biosystem, 5 μg/cm^2^) or collagen (Roche, 5 μg/cm^2^) covered PS. Cells were harvested for analysis by pipetting within the supernatant HSC medium.

### Flow cytometry

Flow cytometry was performed at 0 and 14 DIV. Cells were harvested and washed with 1 ml PBS containing 2 mM EDTA and 5% HSA. 15 μl of FITC-conjugated anti-human CD45 and 15 μl of PE-conjugated anti-human CD34 antibody as well as 10 μl 7AAD were added followed by incubation for 5 min under light exclusion at room temperature (RT). Corresponding isotype controls were prepared similarly using FITC- and PE-conjugated IsoClonic^TM^ control antibodies. All antibodies and dyes were obtained from Beckman Coulter (Stem-Kit Reagents). After washing in PBS, 1x10^4^ cells per sample were analyzed on a FACS Canto™ II (BD Bioscience); data were gated to exclude debris.

For further characterization of HSC surface markers, a panel of antibodies was used: PE-conjugated anti-human CD34 antibody (1:20 diluted), FITC-conjugated anti-human CD49f antibody (1:100 diluted), APC-conjugated anti-human CD90 antibody (1:10 diluted), PE-Cy7-conjugated anti-human CD38 antibody (1:20 diluted; ThermoFisher Scientific) and Alexa Fluor700^®^ anti-human CD45RA antibody (1:50 diluted; BioLegend). Cell suspensions were incubated with antibodies for 30 min at RT under light exclusion. Isotype controls were prepared similarly using corresponding IgG kappa isotype controls (ThermoFisher Scientific & BioLegend). After washing in PBS, 1 μg/ml DAPI (Sigma Aldrich) was added to the samples and whole samples were analyzed on a LSR Fortessa (BD Bioscience); data were gated to exclude debris.

### Fluorescence microscopy

Cells were incubated with 1 μg/ml DAPI (Sigma Aldrich) and 7.5 μM Draq5 (ThermoFisher Scientific) at RT for 10 and 25 min, respectively. Cells were fixed by adding 37% formaldehyde (final 3.7%) into supernatant HSC medium and incubated for 1 h at RT. Next, samples were washed 3x with PBS and incubated for 1 h at RT in blocking solution (BS: 1% BSA, 0.4% Triton-X 100 in PBS). Samples were incubated in anti-human CD34 primary antibody (Santa Cruz Biotechnology, 1:250 in BS) overnight at 4°C. Afterwards, samples were 3x times with PBS and incubated for 2 h at RT in AlexaFlour^®^555-conjugated anti-mouse secondary antibody (Abcam; 1:500 in BS). Finally, samples were washed 3x with PBS before mounting with ProLong™ Gold Antifade Mountant (ThermoFisher Scientific). Z-stack images were taken with a Zeiss Axio Imager microscope equipped with an ApoTome.2 slider (Carl Zeiss) as well as with a Zeiss Axio Scan.Z1 microscope (Carl Zeiss) and analyzed using Zen 2.0 software (Carl Zeiss).

### Colony forming unit (CFU) assay

After expansion on 2D or 3D surfaces/scaffolds, 1x10^4^ cells per condition were plated in individual 35 mm dishes (Greiner) in 2 ml multi-lineage CFU media (0.75 ml FCS (Lonza), 0.5 ml Iscove's Modified Dulbecco's Media (IMDM; Gibco), 0.4 ml IMDM double conc. (Gibco), 0.25 ml 10% BSA (Stemcell; in IMDM), 20 μl Transferin (Sigma; 27 mg/ml in IMDM), 15 μl 0.093% 2-mercaptoethanol (Sigma; in PBS), 10 μl IL3 (Peprotech; 2.5 μg/ml), 10 μl IL6 (Peprotech; 2.5 μg/ml), 10 μl SCF (Peprotech; 2.5 μg/ml), 10 μl GM-CSF (Peprotech; 10 ng/ml), 100 U/ml penicillin and 100 U/ml streptomycin (Lonza), 6 μl/ml L-glutamine (Biochrom; 200mM), 5 μl/ml erythropoietin (Janssen-Cilag GmbH, in IMDM, stock 100 U)) and 400 μl agar (BD Bioscience; 200 mg/10 ml H_2_O) per dish. Colonies were scored after additional 14 DIV. The nature of CFUs: colony-forming unit-granulocyte/erythroid/megakaryocyte/monocyte (CFU-GEMM), colony-forming unit-granulocyte/macrophage (CFU-GM), burst-forming unit-erythroid (BFU-E), colony-forming unit-erythroid (CFU-E), colony-forming unit-macrophage (CFU-M) and colony-forming unit-granulocyte (CFU-G) was scored and confirmed according the quality assurance procedure instruction of the University Hospital Jena.

### Statistical analysis

Results are displayed as mean values ± standard deviation (SD) using GraphPad Prism 8. Statistical significances of data were tested using a two-tailed paired parametric t-test using GraphPad Prism 8 (* p< 0.05; **p<0.01; ***p<0.001).

## Results

**Fig 1:**
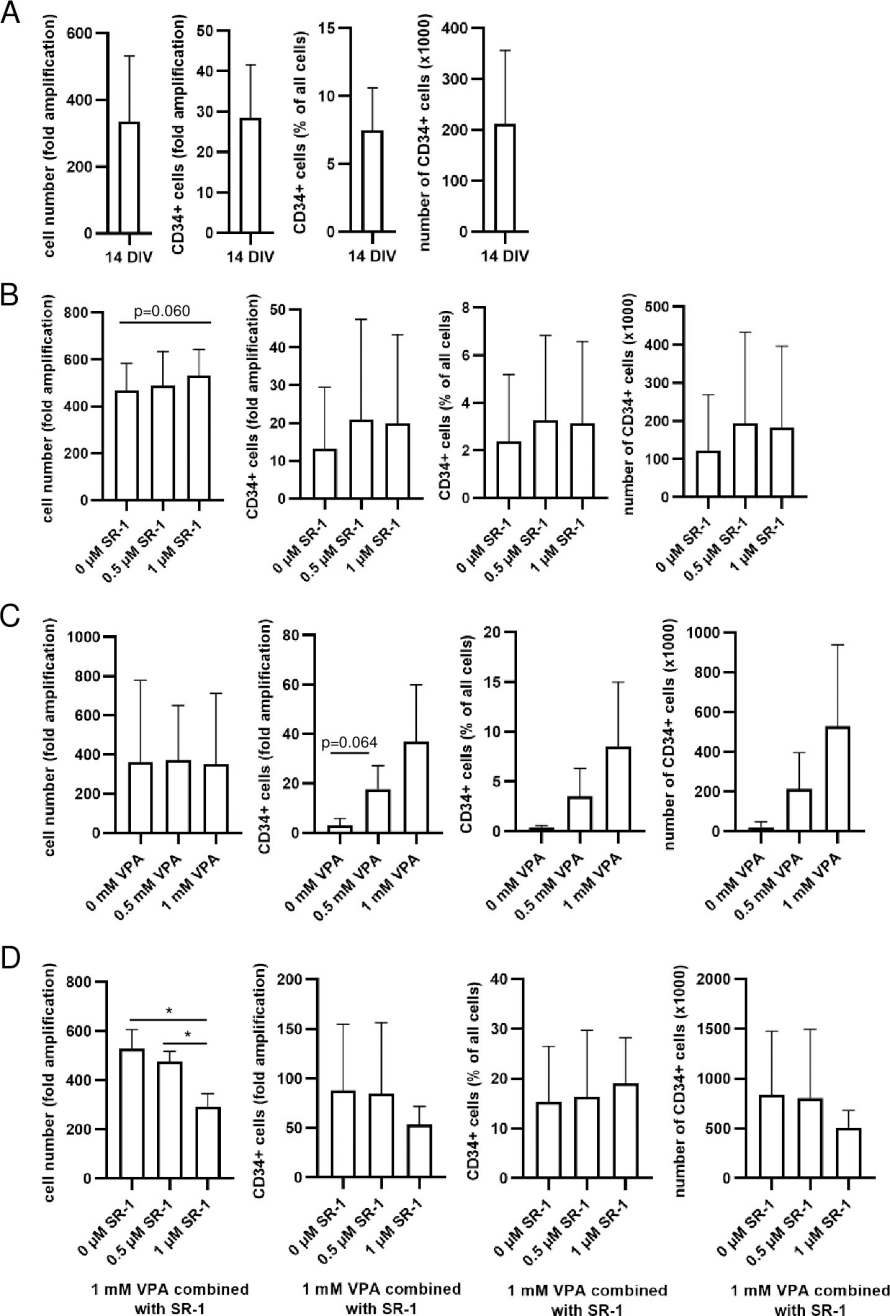
Valproic acid (VPA) and Stemreginin-1 (SR-1)-dependent effects on the growth of human hematopoietic stem cells (HSCs) *in vitro*. Human HSCs were cultured in Stemline II Hematopoetic Stemcell Medium supplemented with 100 ng/ml FLT3, 100 ng/ml SCF, 100 ng/ml TPO and 50 ng/ml IL-3 (HSC medium). CD34+ cells were determined by flow cytometric analyses using an anti-CD34/CD45 mouse monoclonal antibody and 7AAD. (A) Fold amplification of all cells, CD34+ cells as well as percentage and absolute cell number of CD34+ cells after 14 days *in vitro* (DIV) are depicted for three different donors as their mean ± SD. HSCs were treated with 0.5 or 1 μM SR-1 (B), 0.5 or 1 mM VPA (C) or with 1 mM VPA in combination with 0.5 or 1 μM SR-1 (D) for 14 DIV are depicted for three different donors as their mean ± SD. *p<0.05.

First we optimized commonly used HSC culture medium by supplementation with a panel of hematopoietic cytokines (short HSC medium; see methods for further information), which separately have been shown to promote stem cell maintenance *in vitro* [3, 14]. After 14 days *in vitro* (DIV) on commonly used 2D polystyrene (PS; blank cell culture plates), the total cell number and number of CD34+ cells was amplified (Fig 1A). Since we used CD34+ pre-selected cells, their percentage decreased between 0 and 14 DIV; nevertheless, overall numbers of CD34+ cells increased (Fig 1A and S1A Fig) together with their viability (S1B Fig). Hence, the HSC medium proofed to amplify CD34+ cells and to maintain their viability during *in vitro* cultivation.

To further improve CD34+ cell expansion, we tested two compounds, i.e. stemreginin-1 (SR-1) and valproic acid (VPA), which have been described to support HSC proliferation and pluripotency maintenance [3, 14, 15]. SR-1 only modestly affected total cell numbers and CD34+ cell amplification (Fig 1B and S1C Fig). In contrast, VPA enhanced the number as well as the percentage of CD34+ cells in a dose-dependent manner (Fig 1C and S1E Fig). The amplification of CD34+ cells ranged from 3.7-fold without VPA to 37-fold using 1 mM VPA, whereas, the amplification of the total cell number was nearly unaffected by VPA (Fig 1C and S1E Fig). Combinations of SR-1 with 1 mM VPA lowered the amplification of all cells, CD34+ cells and CD34+ cells numbers compared to VPA alone (Fig 1D and S1C Fig), indicating an antagonistic effect between SR-1 and VPA. The viability of CD34+ cells was not affected by SR-1 or VPA treatment regimens (S1D and S1F Fig). In addition, we compared the effects of supplemented cytokines (see methods) with/without VPA on HSC growth during 7 and 14 DIV (Fig 1G and 1H). The cytokines strongly improved the amplification of all cells, CD34+ cells and the number of CD34+ cells. VPA further enhanced these effects and enriched the percentage of CD34+ cells during 14 DIV, however, VPA alone was not sufficient to promote HSC growth. The cell viability after 14 DIV decreased without cytokine supplementation. Thus, both cytokines and VPA were necessary to amplify a viable population of CD34+ cells *in vitro* and we used 1 mM VPA as supplement to the HSC medium for the following experiments.

**Fig 2:**
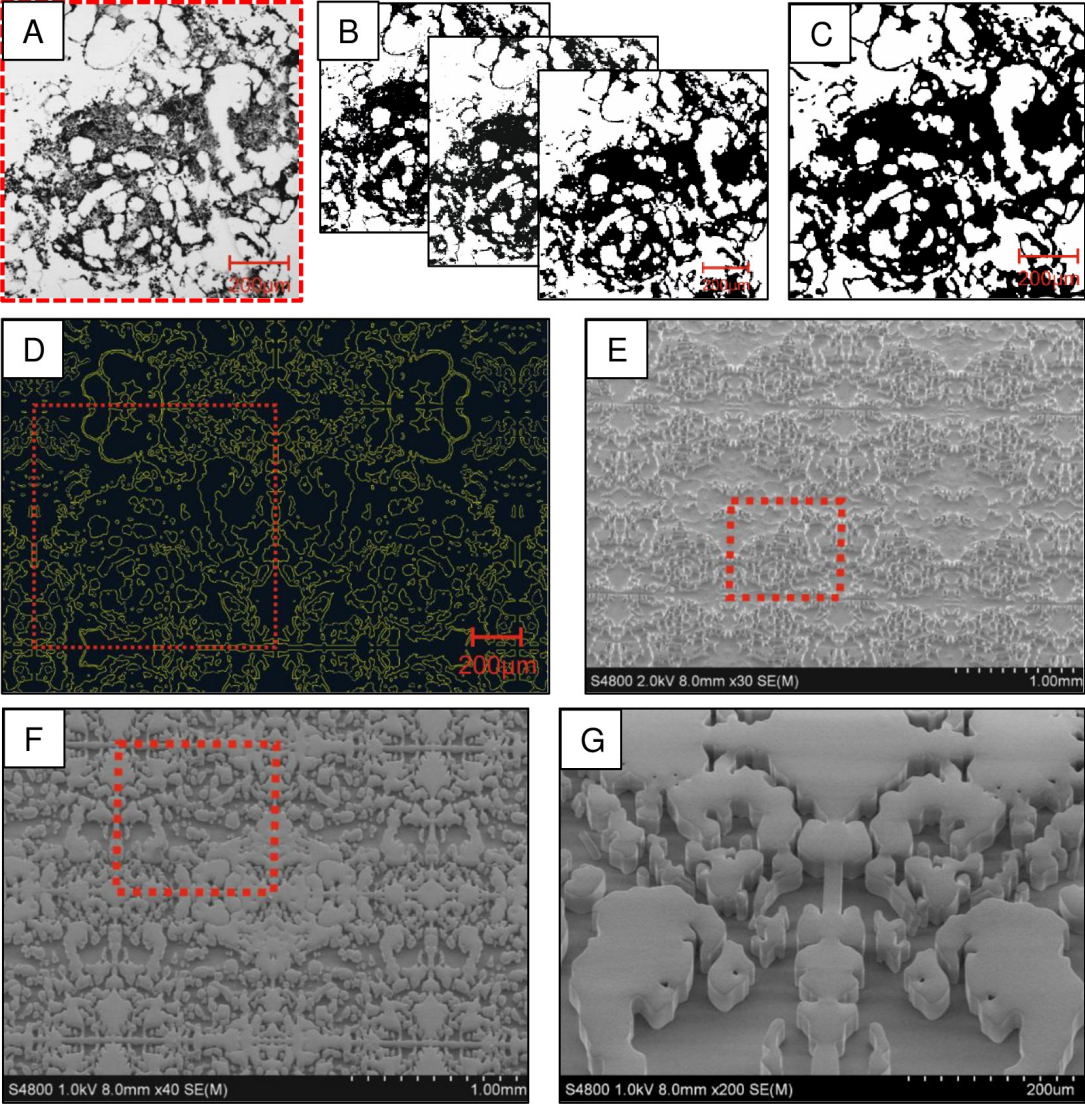
Processing steps to generate a 3D structure mimicking the human HSC niche. (A) The biopsy of the bone marrow (BM) from a human long bone cross section was imaged by laser scanning microscopy. (B) Image processing was done by using edge and threshold algorithms to generation the final vector image for mask translation (C). (D) This vector image was further converted into a photolithographic mask to generate a casting mold through dry etching of a silicon wafer (E). (F-G) Polydimethylsiloxane (PDMS) was casted into the silicon mold to generate 3D PDMS structures. Red squares indicate the initial structural element which was obtained from the BM biopsy.

Next, we developed a 3D PDMS cell culture system based on the BM structure of a human long bone cross section (Fig 2A). The image of the cross section was processed to generate a photolithographic mask (Fig 2B–2D). A casting mold was generated through dry etching of a silicon master to produce 3D PDMS structures (Fig 2E–2G). This 3D scaffold was designed to mimic the stem cell niche *in vitro* and thus, to support their proliferation and to diminish HSC differentiation into lineage-specific progenitor cells. We initially analyzed the cell growth on 3D PDMS scaffolds and cell harvesting efficiency (S2A and S2B Fig) and found that cells grew within the scaffolds and could be completely harvested by pipetting.

**Fig 3:**
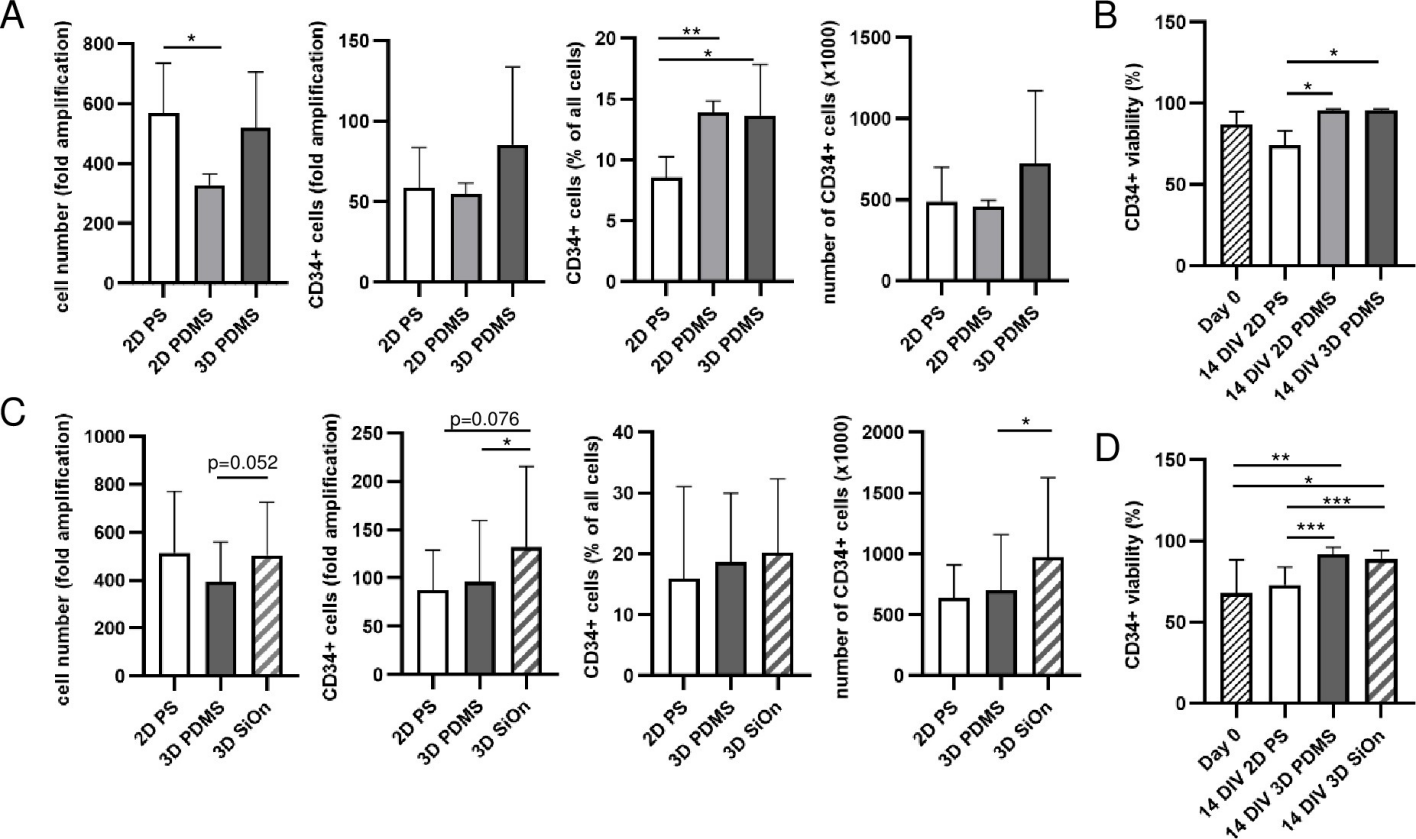
Effects of 2D and 3D polydimethylsiloxane (PDMS) structures on human HSC cultivation. Human HSCs were cultured for 14 DIV in HSC medium supplemented with 1 mM VPA on SiOn-covered and uncovered 2D PDMS or 3D PDMS scaffolds. CD34+ cells were determined by flow cytometric analyses using a combination of anti-CD34/anti-CD45 mouse monoclonal antibody and 7AAD. (A) The amplification of all cells and CD34+ cells, the percentage and absolute number of CD34+ cells as well as their viability (B) are depicted for four different donors as their mean ± SD. (C) The amplification of all cells and CD34+ cells, the percentage and the absolute number of CD34+ cells as well as their viability (D) are depicted for nine different donors as their mean ± SD. *p<0.05, **p<0.01, ***p<0.01.

Then, we cultivated HSCs on 3D PDMS, 2D PS and unstructured 2D PDMS as experimental controls within the HSC medium. After 14 DIV, 3D PDMS increased total cell numbers more efficiently than 2D PDMS, but did not offer benefits compared to 2D PS (Fig 3A and S2C Fig). However, the amplification, percentage and the number of CD34+ cells increased during 14 DIV on 3D PDMS scaffolds (*p<0.05) compared to 2D PDMS and 2D PS (Fig 3A and S2C Fig). The CD34+ cell viability was higher on PDMS scaffolds compared to 2D PS (*p<0.05) and to 0 DIV (Fig 3B). In addition, we analyzed the growth of CD34+ cells on 3D PDMS in the absence of VPA (S2D–S2F Fig). The 3D PDMS scaffolds already offered benefits compared to 2D PS (compare Fig 1C), however, we found that 1 mM VPA was necessary to achieve optimal viability, amplification, percentage and total number of CD34+ cells after 14 DIV (S2E and S2F Fig). Therefore, 3D PDMS in combination with 1 mM VPA in the HSC medium appears to be most suitable for HSC *in vitro* cultivation.

Human HSCs are semi-adherent, which is important for their homing and their attachment in the BM compartment [16]. PDMS has a hydrophobic surface, which might influence the attachment of human HSCs *in vitro* [17]. Therefore, we covered 3D PDMS structures with silicon oxide (SiOn)-based Aquacer^®^-covering to reduce the hydrophobicity. The amplification of all cells after 14 DIV on SiOn-covered 3D PDMS (3D SiOn) was comparable to 2D PS, but improved compared to 3D PDMS (Fig 3C). 3D SiOn enhanced the amplification of CD34+ cells (*p<0.05 compared to 3D PDMS) and absolute number of CD34+ cells compared to 3D PDMS and 2D PS (*p<0.05 compared to 2D PS), whereas the percentages of CD34+ cells was comparable between 3D PDMS and 3D SiOn (Fig 3C and S2G Fig). Both uncovered and SiOn-covered 3D PDMS scaffolds improved CD34+ cell viability compared to 2D PS after 14 DIV (***p<0.001) as well as compared to day 0 (**p<0.01 for 3D PDMS and *p<0.05 for 3D SiOn) (Fig 3D). Taken together, SiOn-covering of PDMS scaffolds further improved the specific amplification of CD34+ cells with a clear advantage of the 3D system over 2D PS.

**Fig 4:**
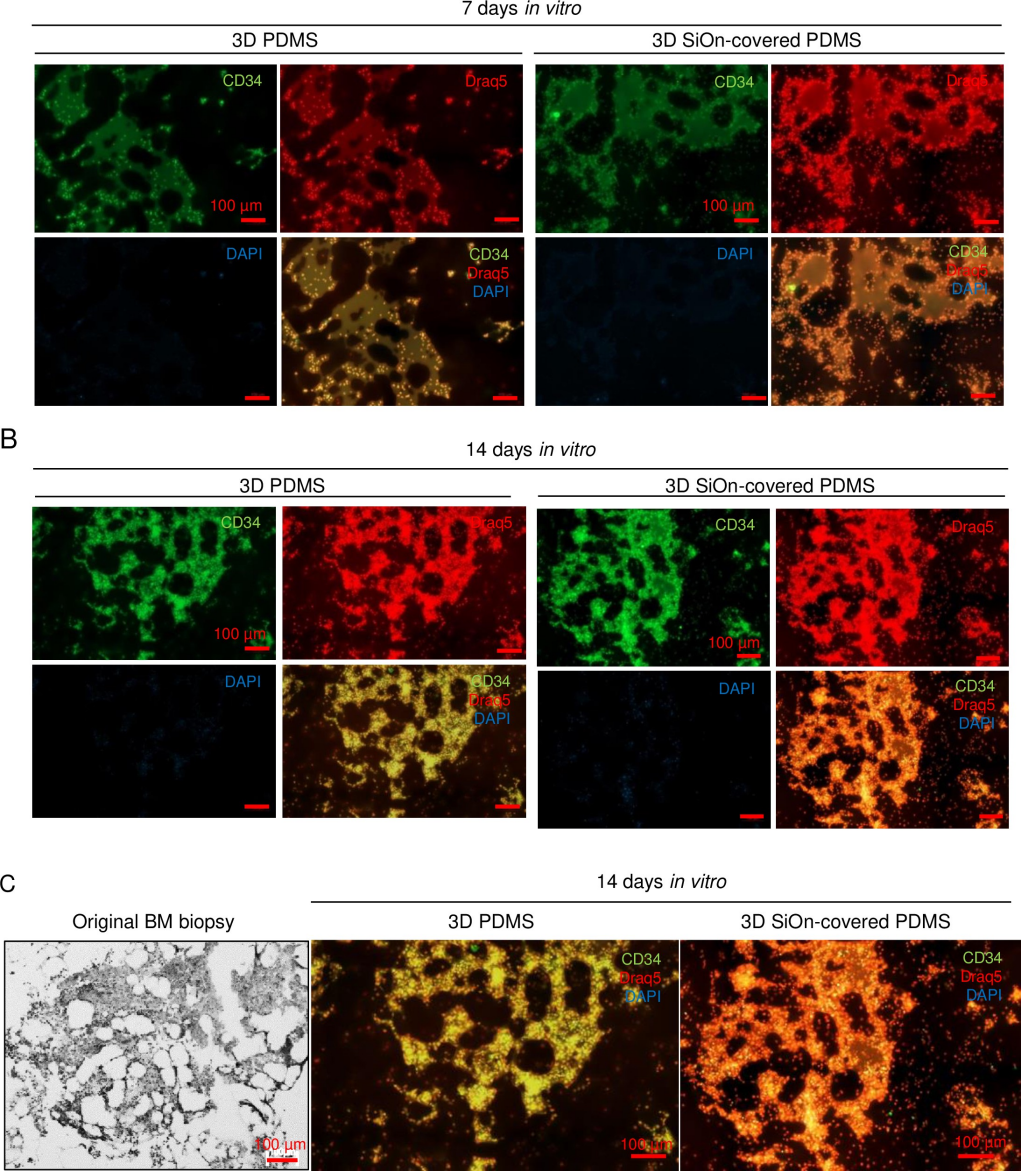
Microscopy of human HSCs cultured on SiOn-covered and uncovered 3D PDMS structures. Human HSCs were cultured in HSC medium supplemented with 1 mM VPA on SiOn-covered and uncovered 3D PDMS scaffolds. All cell nuclei were stained with Draq5 (red) and dead cells were stained by DAPI uptake (blue) shortly before fixation. CD34+ cells were immune-stained after fixation with a primary anti-CD34 antibody in combination with an Alexa Fluor555-conjugated secondary antibody (green). Z-stack images of whole scaffolds were taken on an Axio Scan.Z1 Slide Scanner microscope in Z-stacks after 7 (A) and 14 (B) DIV. The pictures show reconstructions of extended focus images 2D projection of multiple Z-stacks and are representative for three independent experiments. Full images of A and B are shown in S3 Fig. Comparison between of the initial BM biopsy and SiOn-covered and uncovered 3D PDMS structures after 14 DIV are depicted in (D; pictures are identical to the ones shown in Fig 4A and 4B).

To address if cells would grow within the cavities of the scaffolds or on top, and the influence of SiOn-covering, we analyzed 3D scaffolds after 7 and 14 DIV by fluorescence microscopy (Fig 4 and S3 Fig). Full scaffolds were analyzed using a whole slide scanner, allowing a stepwise magnification from an overview perspective up to single-cell level on every position of the structure (S3A and S3B Fig). Additionally, detailed pictures from the scaffolds were taken (S3C Fig). After 7 DIV, clearly more cells were visible on 3D SiOn than on 3D PDMS. CD34+ cells were found mostly within the cavities of uncovered 3D PDMS, whereas the SiOn-covering enabled cells to settle down on the surface of the PDMS structures, too (Fig 4A and S3 Fig). Intriguingly, the cells on the surface had a lower CD34+ fluorescence signal compared to the ones within the cavities. After 14 DIV, cell densities on 3D PDMS closely reached the ones seen on 3D SiOn (Fig 4B and S3 Fig). Cells were still mostly found within the cavities; however, they were also found on the surface of both 3D PDMS scaffolds (Fig 4B and S3 Fig). Again, the cells on the surface of the scaffolds had a lower or even no CD34+ fluorescence signal. The amount of dead, DAPI positive cells after 7 and 14 DIV was low and comparable between 3D SiOn and 3D PDMS scaffolds and increased during time (Fig 4A and 4B and S3 Fig). Since human HSCs are semi-adherent, we asked if CD34+ cells were located on the sidewalls of the cavities. In case of 3D PDMS, CD34+ cells seemed to accumulate in the center of the cavities but were almost not located near the sidewalls. The more hydrophilic surface of 3D SiOn allowed cells to settle down along the sidewalls of the cavities, too (Fig 4A and 4B and S3 Fig). We compared the original biopsy of the human BM, which was used to create our 3D scaffolds, with pictures of SiOn-covered and uncovered 3D PDMS structures after 14 DIV (Fig 4C). Within the biopsy, cells grew in the cavities of the BM. Taken the above-mentioned differences between 3D SiOn and 3D PDMS into account, cells indeed grew the same way in our 3D PDMS scaffolds like in the human BM (Fig 4C). These results indicate that cells preferentially use the cavities of the 3D PDMS scaffolds, that SiOn-covering improves their attachment and that 3D PDMS scaffolds are suitable to mimic the human HSC niche.

**Fig 5:**
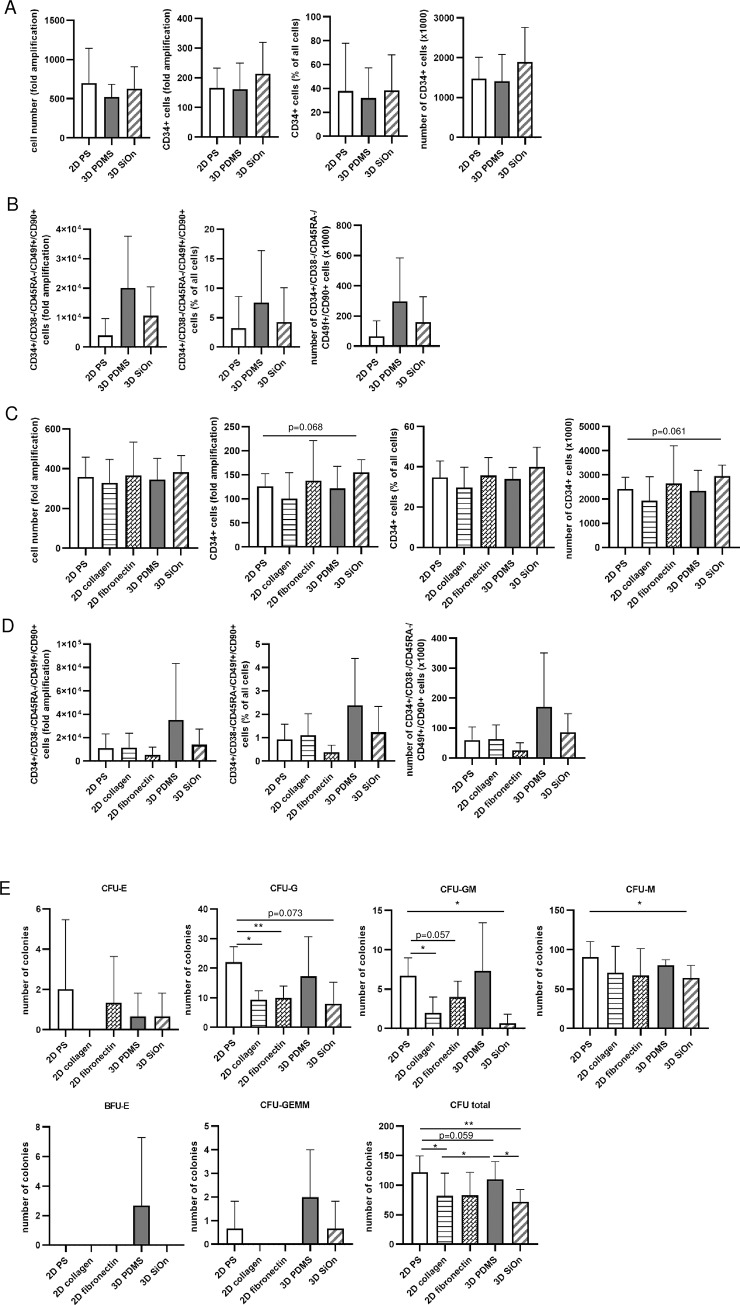
Characterization of naïve human HSCs grown on SiOn-covered and uncovered 3D PDMS and collagen- or fibronectin-covered and uncovered PS. Human HSCs were cultured for 14 DIV in HSC medium supplemented with 1 mM VPA on uncovered 2D PS, 3D PDMS, collagen- or fibronectin-covered 2D PS as well as SiOn-covered 3D PDMS. CD34+ and CD34+/ CD38-/ CD45RA-/ CD49f+/ CD90+ cells were determined by flow cytometric analyses using a combination of specific antibodies and vital cells were selected using DAPI. CFUs were counted after additional 14 days of incubation in multi-lineage CFU medium. The amplification of all cells and CD34+ cells, the percentage and absolute number of CD34+ cells are depicted for three different donors (A, C) as their mean ± SD. The amplification, percentage and the absolute cell number of CD34+/ CD38-/ CD45RA-/ CD49f+/ CD90+ cells are depicted for three different donors (B, D) as their mean ± SD. Total numbers of CFU per 1x10^4^ seeded human HSCs are depicted for three different donors (E) as their mean ± SD. Colony-forming unit-granulocyte/erythroid/megakaryocyte/monocyte (CFU-GEMM), colony-forming unit-granulocyte/macrophage (CFU-GM), burst-forming unit-erythroid (BFU-E), and colony-forming unit-erythroid (CFU-E), colony-forming unit-macrophage (CFU-M), colony-forming unit-granulocyte (CFU-G). *p<0.05, **p<0.01.

Although CD34 is generally used as a marker to discriminate between stem cells and differentiated cells, it is alone not suitable to label naïve HSCs. For a more detailed characterization of the CD34+ cells after 14 DIV, we introduced a panel of additional cell surface markers for flow cytometry according to Laurenti and Goettgens [18] (Data were gated as shown in S4 Fig). In line with our previous results (Fig 3C), 3D SiOn improved the amplification, the percentage and total number of CD34+ cells compared to 2D PS and 3D PDMS (Fig 5A). However, if additional markers for HSC immaturity, i.e. CD38-, CD45RA-, CD49f+ and CD90+ were taken into account, 3D PDMS showed the best amplification of cells, increase in total cell number and percentage of CD34+/CD38-/CD45RA-/CD49f+/CD90+ cells after 14 DIV (Fig 5B and S5A Fig). Thus, uncovered 3D PDMS seemed to be the better cell culture system to amplify naïve HSCs *in vitro*, although the total number of CD34+ cells was lower compared to SiOn-covered 3D PDMS.

Finally, we analyzed the actual pluripotency and repopulation potential of the HSCs cultured on 3D PDMS scaffolds in comparison to well-established fibronectin-covered 2D PS (2D fibronectin) [19], uncovered 2D PS and collagen-covered 2D PS (2D collagen) by flow cytometry and CFU assays (Fig 5C–5E). 2D collagen proofed to be disadvantageous for the amplification of CD34+ cells (Fig 5C); 2D fibronectin improved the amplification, the percentage and total number of CD34+ cells compared to uncovered 2D PS and 3D PDMS. 3D SiOn showed the highest amplification of CD34+ cells, percentage of CD34+ cells and total number of CD34+ cells (Fig 5C and S5B Fig). However, 3D PDMS showed again the highest amplification, total number and percentage of CD34+/CD38-/CD45RA-/CD49f+/CD90+ cells (Fig 5D and S5B Fig). Therefore, 3D PDMS scaffolds improved the amplification of immature HSCs compared to commonly used uncovered 2D PS, 2D collagen and 2D fibronectin [19–23].

To assess the clonogenic potential of the expanded cells from different surfaces/scaffolds, colony forming unit (CFU) assays were performed (Fig 5E, S6 Fig). Thereby, colony-forming unit-erythroid (CFU-E) are clonogenic progenitors containing clusters of hemoglobinized erythroblasts presenting more mature erythroid progenitors with less proliferative capacity. In contrast, burst-forming unit-erythroid (BFU-E) are primitive erythroid progenitors with high proliferative capacity. Colony-forming unit-granulocyte (CFU-G) are clonogenic progenitors of granulocytes that give rise to a homogeneous population of eosinophils, basophils or neutrophils, whereas colony-forming unit-macrophage (CFU-M) are clonogenic progenitors of macrophages that give rise to a homogenous population of macrophages. Colony-forming unit-granulocyte/macrophage (CFU-GM) are progenitors that give rise to colonies containing a heterogeneous population of macrophages and granulocytes with a morphology similar to CFU-M and CFU-G. Colony-forming unit-granulocyte/erythroid/megakaryocyte/monocyte (CFU-GEMM) are multi-lineage progenitors that give rise to erythroid, granulocyte, macrophage and megakaryocyte lineages, reflecting the immaturity of the cells [24]. Cells that were expanded on 2D PS yielded highest total colony counts compared with 2D collagen (*p<0.05) and 2D fibronectin as well as 3D PDMS and 3D SiOn (**p<0.01) and showed the highest number of CFU-E, CFU-M and CFU-G. Whereas, cells grown on 3D PDMS yielded significantly more total colony counts than cells grown on 2D collagen (*p<0.05) and 3D SiOn (*p<0.05). Although, cells grown on 2D PS showed more CFU-GM than cells amplified on 3D SiOn (*p<0.05), 2D collagen (*p<0.05) or 2D fibronectin, the highest number of CFU-GM yielded cells expanded on 3D PDMS. We just found BFU-E colonies and most of CFU-GEMM colonies in cells grown on 3D PDMS. Of note, cells forming BFU-E and CFU-GEMM colonies are characterized by a high proliferative capacity and immaturity. This confirms the results accessed by flow cytometry that cells grown on 3D PDMS have the highest number of immature (LT)-HSCs after 14 DIV (Fig 5A–5D). Cells expanded on 2D PS or 3D SiOn yielded less CFU-GEMM and cells expanded on 2D fibronectin or 2D collagen showed no CFU-GEMM.

Taken together, we were able to strongly improve HSC proliferation as well as pluripotency maintenance by optimizing first, the HSC *in vitro* culture medium and second, the cell culture environment with 3D PDMS scaffolds by biomimetic reconstruction of the natural human BM.

## Discussion

HSCT is the most effective treatment for a broad spectrum of hematopoietic malignancies and several non-malignant congenital and acquired disorders. Since there is often a disparity between the amount of HSCs needed by the patient and those available from the donor, an effective *in vitro* amplification prior to HSCT is required, but not yet sufficiently realized [3, 5]. We aimed to find an environment for human HSCs that increases their proliferation, maintains their pluripotency and ability for self-renewal by reducing their differentiation into lineage-specific progenitor cells and offering optimal growth conditions. Since we used primary human material for our experiments, the number of cells, amplification rates and number of CD34+ cells as well as the percentage of CD34+ cells varied between the different donors.

First, we optimized the HSC medium to increase the numbers of CD34+ cells after 14 DIV (Fig 1A). In accordance with Chaurasia, *et al* [14], we found that VPA improved stem cell maintenance and proliferation of CD34+ cells (Fig 1C). VPA belongs to the group of histone deacetylase inhibitors (HDACi) and is therefore an epigenetic modifier. Additionally, VPA is known to inhibit glycogen synthase kinase 3 beta (GSK3B) functions by inducing its phosphorylation on Ser9, which activates Wnt target genes accompanied by an up-regulation of homeobox B4 protein (HoxB4). Both, HoxB4 and Wnt are direct stimulators of HSC proliferation and their potential in self-renewal [25, 26].

The arylhydrocarbon receptor antagonist SR-1 is described to enhance HSC expansion and to support hematopoietic recovery after transplantation of human cord blood (CB)-derived CD34+ cells [15, 27]. In contrast, we observed only modest effects of SR-1 on the expansion of CD34+ cells derived from mobilized peripheral blood (mPB) *in vitro* (Fig 1B). The combination of SR-1 with VPA even decreased the amplification of CD34+ cells compared to VPA alone (Fig 1D). This could be due to different origin of cells, different cytokine composition in the cultivation medium and different SR-1 concentrations in our experimental setup compared to the studies by Wagner, *et al* and Dahlberg, *et al* [15, 27]. Additionally, SR-1 seems to have a narrow spectrum of action and requires distinct conditions to reach its activity. Boitano, *et al* [28] reported that SR-1 did not induce proliferation of CB- and mPB-derived CD34+ cells *in vitro* in the absence of specific cytokines. Thus, we cannot generally exclude SR-1 as a suitable compound to improve the amplification of HSCs *in vitro*; however, VPA appeared to be better suited to maintain stem cell character and to increase the proliferation of CD34+ cells in our case.

Human HSCs functions and behavior *in vivo* strongly depend on their microenvironment, the so-called stem cell niche. Besides various interactions of HSCs with other cells types in the BM, the topological structures of the stem cell niche seem to be important for specific HSC differentiation and stem cell potential maintenance [6–9]. PS is currently the state-of-the-art material for cell cultivation; however, the range of PS-based microscale three-dimensional cell cultivation systems is limited. Thermoplastics such as PDMS together with soft lithographic methods offer rapid prototyping of micro- and nanoscale devices to construct BM-like 3D scaffolds [29]. Hence, we created an artificial 3D PDMS structure mimicking the human BM (Fig 2). We could show that 3D PDMS systems enhanced the amplification, total number and viability of CD34+ cells after 14 DIV, compared to conventional 2D PS (Fig 3A and 3B). Another benefit of 3D PDMS, compared to other materials used for 3D scaffolds, such as 3D gels, foam, ceramic structures or structures made out of natural bone material [30], is the upward accessible surface and optical clarity of the material. This allows microscopic analysis, cell treatment and passaging during *in vitro* culture as well enables simple harvesting and reuse of the expanded HSCs for other purposes like transplantation.

In the next step, surface hydrophobicity of PDMS structures was decreased to offer better attachment of cells. SiOn-covering enhanced the amplification of CD34+ cells (Fig 3C and 3D). However, by taking further HSC surface markers, i.e. CD38-, CD90+, CD49f+ and CD45RA-, into account, we found that this subset of HSCs preferentially grew on the more hydrophobic surface of uncovered 3D PDMS (Fig 5A and 5B). These cell surface markers reflect the maturity and differentiation of human HSCs more accurately than CD34+ alone and are indicative for immature long-term HSCs (LT-HSCs). LT-HSCs are important for steady-state hematopoiesis up to several weeks after HSCT since these immature stem cells have a highly conserved pluripotent character and capacity for self-renewal, whereas CD34+ progenitors are important for a quick hematopoietic reconstitution after HSCT [18, 31].

Fibronectin and collagen, both extracellular matrix proteins, are widely used in cell culture systems to support cell growth by mimicking the *in vivo* cell environment more closely than uncovered PS [9, 32]. Fibronectin-covering is well-established and commonly used for *in vitro* amplification of human HSCs, whereas collagen-covering is used for *in vitro* cultivation of human mesenchymal stem cells but also tested for *ex vivo* HSC expansion. Intriguingly, uncovered 3D PDMS showed higher amplification rates of CD34+/CD38-/CD90+/CD49f+/CD45RA- LT-HSCs, compared to 3D SiOn, 2D collagen and 2D fibronectin (Fig 5D). A possible explanation for the effect of SiOn-covered PDMS or fibronectin-covered PS could be that both facilitate the attachment of HSCs and thus, induce a better growth and amplification in parallel to increased differentiation into HSPCs.

To predict the actual clonogenic potential of the amplified cells from different scaffolds, we performed CFU assays (Fig 5E and S6 Fig). Cells grown on 3D PDMS showed the highest numbers of CFU-GEMM and BFU-E (Fig 5E). Therefore, 3D PDMS seems to the best scaffold to preserve the multi-lineage capacity of HSCs during *in vitro* amplification. Cells cultivated on other surfaces/scaffolds showed lower number of CFU-GEMM and BFU-E and hence, proofed to be inferior to 3D PDMS. This confirms our previous results from flow cytometric analyses that the highest numbers of immature HSCs were also found on 3D PDMS (Fig 5B and 5D) and we conclude that uncovered 3D PDMS offers optimal conditions to amplify human LT-HSCs *in vitro*.

Since the 3D PDMS structures are transparent, we were able to analyze in which region HSCs were preferentially located and which influence the surface covering had on their location. We observed already after 7 DIV clearly more cells in general as well as CD34+ cells in case of SiOn-covered 3D PDMS than on uncovered 3D PDMS. After 14 DIV, cell densities on uncovered 3D PDMS scaffolds reached close to densities on 3D SiOn (Fig 4 and S3 Fig). In line with the results of our FACS analysis (Figs 3 and 5) this suggests, that a hydrophilic surface offers better initial attachment and growths of CD34+ cells after seeding. Additionally, SiOn-covering allows cells to grow outside the cavities on top of the structures and on the cavities sidewalls compared to uncovered 3D PDMS (Fig 4 and S3 Fig). Cells, which grew outside of the cavities, had a lower or even no CD34+ signal, indicating a loss of stem cells character and differentiation. This confirmed our observations by flow cytometry (Figs 3 and 5) that 3D SiOn enhanced cell growth, but HSC differentiation, too, and that immature HSC were better preserved on 3D PDMS.

It remains to be elucidated which repopulating potential the HSCs amplified in this novel *in vitro* cultivation system offer *in vivo*. Nevertheless, by establishing a 3D scaffold according to the human BM, we created a platform mimicking the natural niche of human HSC. 3D PDMS is suitable to amplify human HSC *in vitro* and to support their vitality, pluripotency as well as their ability for self-renewal. In combination with the optimized HSC medium supplemented with VPA, this novel approach may help to further improve HSCT by enabling an effective *in vitro* expansion of HSCs prior to clinical applications.

## Supporting information

S1 Fig Valproic acid (VPA) and Stemreginin-1 (SR-1)-dependent effects on the growth of human hematopoietic stem cells (HSCs) *in vitro*. The number, percentage and the viability of CD34+ cells was determined by flow cytometric analyses using a combination of anti-CD34/anti-CD45 mouse monoclonal antibody and 7AAD at day 0 and 14 of *in vitro* culture. The percentage and absolute number of CD34+ cells after 0 days *in vitro* (DIV) (A) as well as the corresponding viability of CD34+ cells after 0 and 14 DIV (B) are depicted for three different donors as their mean ± SD. The percentage and absolute number of CD34+ cells after 0 DIV (C) are depicted for three different donors as their mean ± SD. The corresponding viability of CD34+ cells after 0 and 14 DIV in the presence of 0.5 or 1 μM SR-1 as well as of 1 mM VPA in combination with 0.5 or 1 μM SR-1 (D) is depicted for three different donors as their mean ± SD. The percentage and absolute number of CD34+ cells after 0 DIV (E) are depicted for three different donors as their mean ± SD. The corresponding viability of CD34+ cells after 0 and 14 DIV in the presence of 0.5 or 1 mM VPA (F) is depicted for three different donors as their mean ± SD. (G) The percentage, viability and absolute number of CD34+ cells after 0 DIV are depicted for seven different donors as their mean ± SD. (H) The corresponding amplification of all cells and of CD34+ cells as well as the percentage, viability and absolute number of CD34+ cells are depicted for seven different donors as their mean ± SD.
(PPTX)

S2 Fig Harvest efficiency of human HSCs grown on 3D PDMS structures and effects of 2D and 3D PDMS structures and silicon oxide covering on human HSC cultivation. A-B: Human HSCs were cultured in HSC medium supplemented with 1 mM VPA on uncovered 3D PDMS scaffolds for 14 DIV. All cell nuclei were stained with Draq5 (red) and dead cells were stained by DAPI uptake (blue) shortly before fixation. Z-stack images of whole scaffolds were taken on an Axio Scan.Z1 Slide Scanner microscope in Z-stacks before (A) and after (B) harvesting. The pictures show reconstructions of extended focus images 2D projection of multiple Z-stacks and are representative for three independent experiments. C-G: The absolute number, percentage and the viability of CD34+ cells was determined by flow cytometric analyses using a combination of anti-CD34/anti-CD45 mouse monoclonal antibody and 7AAD at days 0 and 14 of *in vitro* culture. (C) The percentage and absolute cell number of CD34+ cells after 0 DIV are depicted for four different donors as their mean ± SD. (D) The percentage, the absolute number and viability of CD34+ cells after 0 DIV are depicted for three different donors as their mean ± SD. (E) Human HSCs were cultured in HSC medium supplemented without or with 0.5 or 1 mM VPA on uncovered 3D PDMS scaffolds for 14 DIV. The fold amplification of all cells and CD34+ cells, the percentage and the absolute cell number of CD34+ cells are depicted for three different donors as their mean ± SD. (F) The corresponding viability of CD34+ cells after 14 DIV in the presence of 0.5 or 1 mM VPA is depicted for three different donors as their mean ± SD. (G) The percentage and absolute number of CD34+ cells after 0 DIV are depicted for nine different donors as their mean ± SD.
(PPTX)

S3 Fig Microscopy of human HSCs cultured on SiOn-covered and uncovered 3D PDMS structures. Human HSCs were cultured in HSC medium supplemented with 1 mM VPA on SiOn-covered and uncovered 3D PDMS scaffolds. All cell nuclei were stained with Draq5 (red) and dead cells were stained by DAPI uptake (blue) shortly before fixation. CD34+ cells were immune-stained after fixation with a primary anti-CD34 antibody in combination with an Alexa Fluor555-conjugated secondary antibody (green). Z-stack images of whole scaffolds were taken on an Axio Scan.Z1 Slide Scanner microscope in Z-stacks after 7 (A) and 14 (B) DIV. The pictures show reconstructions of extended focus images 2D projection of multiple Z-stacks and are representative for three independent experiments. Detail images of selected areas were taken with an Axio Imager equipped with an ApoTome.2 slider ApoTome microscope for optical sectioning in Z-stacks after 7 and 14 DIV (C). The pictures show orthogonal 2D projection of multiple Z-stacks and are representative for three independent experiments.
(PPTX)

S4 Fig Gating scheme according to Fig 5. Cells were stained with DAPI and monoclonal antibodies conjugated with different fluorophores against CD34, CD38, CD49f, CD45RA and CD90. Prior to flow cytometric analyses of CD34+ and CD34+/ CD38-/ CD45RA-/ CD49f+/ CD90+ cells, a doublet discrimination was done and vital cells were selected using DAPI. According to the IgG control to every antibody (not shown), gates were set and distinct populations were determined.
(PPTX)

S5 Fig Characterization of naïve human HSCs grown on SiOn-covered and uncovered 3D PDMS and collagen- or fibronectin-covered and uncovered PS. CD34+ and CD34+/ CD38-/ CD45RA-/ CD49f+/ CD90+ HSC cells after 0 DIV were determined by flow cytometric analyses using a combination of specific antibodies and vital cells were selected using DAPI. The percentage and absolute number of CD34+ cells as well as the percentage and the absolute number of CD34+/ CD38-/ CD45RA-/ CD49f+/ CD90+ cells are depicted for three different donors as their mean ± SD (A and B show independent experiments).
(PPTX)

S6 Fig Morphological evaluation of the colonies. A: Burst-forming unit-erythroid (BFU-E), B: Colony-forming unit-granulocyte/erythroid/megakaryocyte/monocyte (CFU-GEMM), C: Colony-forming unit-erythroid (CFU-E), D: Colony-forming unit-macrophage (CFU-M), E: Colony-forming unit-granulocyte (CFU-G), F: Colony-forming unit-granulocyte/macrophage (CFU-GM). Images were examined using a Zeiss AxioVert 40 CFL microscope and an AxioCam MRc 5 (Carl Zeiss) after 14 days of incubation in multi-lineage CFU medium.
(PPTX)
