# Supplementary material for: Biomimetic reconstruction of the hematopoietic stem cell niche for in vitro amplification of human hematopoietic stem cells
Source: PLoS One. 2020 Jun 22;15(6):e0234638. doi: 10.1371/journal.pone.0234638 (PMC7307768; doi:10.1371/journal.pone.0234638)
Supplement: S1 Fig — The number, percentage and the viability of CD34+ cells was determined by flow cytometric analyses using a combination of anti-CD34/anti-CD45 mouse monoclonal antibody and 7AAD at day 0 and 14 of in vitro culture. The percentage and absolute number of CD34+ cells after 0 days in vitro (DIV) (A) as well as the corresponding viability of CD34+ cells after 0 and 14 DIV (B) are depicted for three different donors as their mean ± SD. The percentage and absolute number of CD34+ cells after 0 DIV (C) are depicted for three different donors as their mean ± SD. The corresponding viability of CD34+ cells after 0 and 14 DIV in the presence of 0.5 or 1 μM SR-1 as well as of 1 mM VPA in combination with 0.5 or 1 μM SR-1 (D) is depicted for three different donors as their mean ± SD. The percentage and absolute number of CD34+ cells after 0 DIV (E) are depicted for three different donors as their mean ± SD. The corresponding viability of CD34+ cells after 0 and 14 DIV in the presence of 0.5 or 1 mM VPA (F) is depicted for three different donors as their mean ± SD. (G) The percentage, viability and absolute number of CD34+ cells after 0 DIV are depicted for seven different donors as their mean ± SD. (H) The corresponding amplification of all cells and of CD34+ cells as well as the percentage, viability and absolute number of CD34+ cells are depicted for seven different donors as their mean ± SD. (PPTX) [file pone.0234638.s001.pptx]

## Slide 1
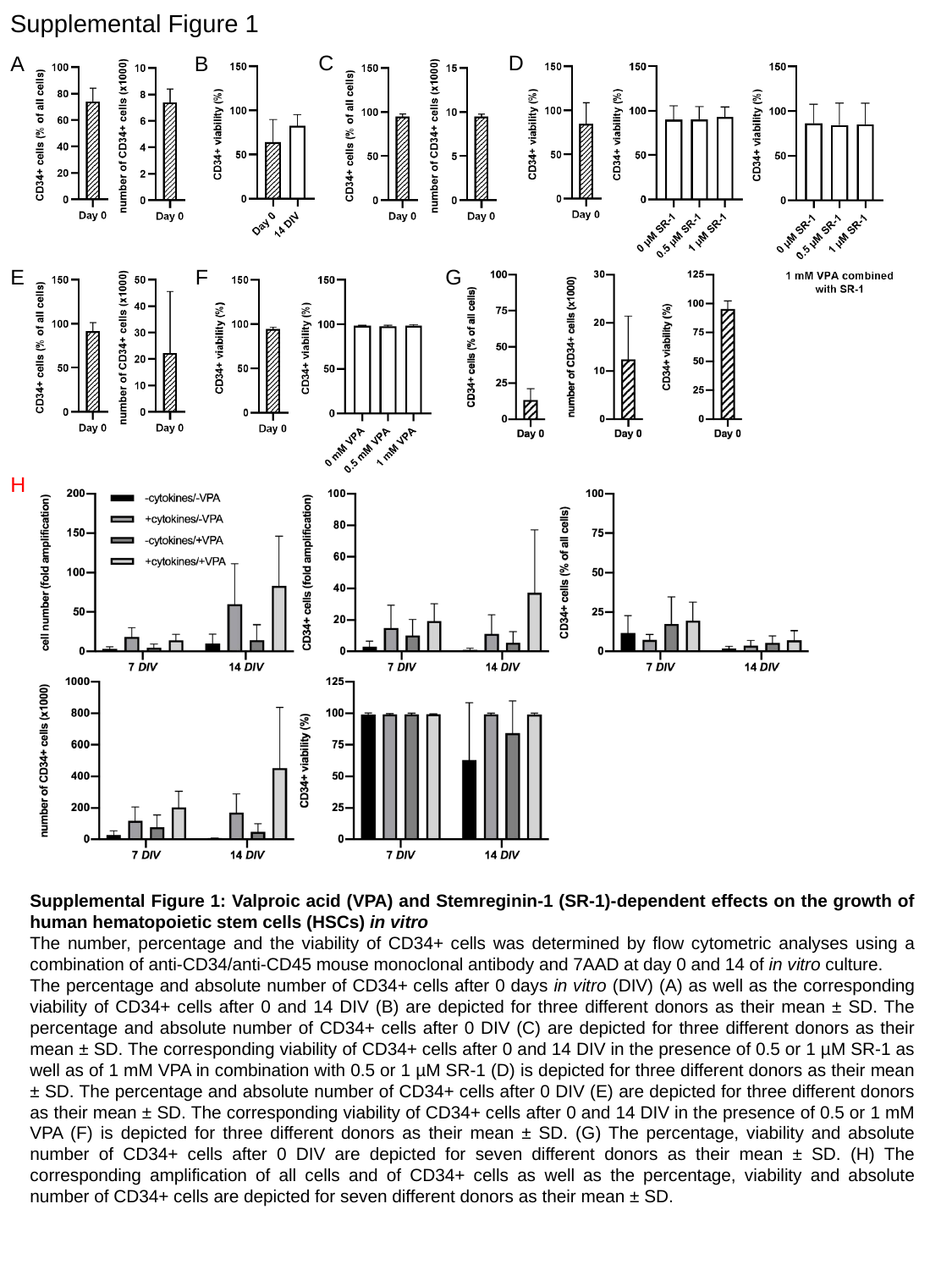

Supplemental Figure 1
C
D
B
A
E
F
G
H
Supplemental Figure 1: Valproic acid (VPA) and Stemreginin-1 (SR-1)-dependent effects on the growth of human hematopoietic stem cells (HSCs) in vitro
The number, percentage and the viability of CD34+ cells was determined by flow cytometric analyses using a combination of anti-CD34/anti-CD45 mouse monoclonal antibody and 7AAD at day 0 and 14 of in vitro culture.
The percentage and absolute number of CD34+ cells after 0 days in vitro (DIV) (A) as well as the corresponding viability of CD34+ cells after 0 and 14 DIV (B) are depicted for three different donors as their mean ± SD. The percentage and absolute number of CD34+ cells after 0 DIV (C) are depicted for three different donors as their mean ± SD. The corresponding viability of CD34+ cells after 0 and 14 DIV in the presence of 0.5 or 1 µM SR-1 as well as of 1 mM VPA in combination with 0.5 or 1 µM SR-1 (D) is depicted for three different donors as their mean ± SD. The percentage and absolute number of CD34+ cells after 0 DIV (E) are depicted for three different donors as their mean ± SD. The corresponding viability of CD34+ cells after 0 and 14 DIV in the presence of 0.5 or 1 mM VPA (F) is depicted for three different donors as their mean ± SD. (G) The percentage, viability and absolute number of CD34+ cells after 0 DIV are depicted for seven different donors as their mean ± SD. (H) The corresponding amplification of all cells and of CD34+ cells as well as the percentage, viability and absolute number of CD34+ cells are depicted for seven different donors as their mean ± SD.
