# Supplementary material for: Biomimetic reconstruction of the hematopoietic stem cell niche for in vitro amplification of human hematopoietic stem cells
Source: PLoS One. 2020 Jun 22;15(6):e0234638. doi: 10.1371/journal.pone.0234638 (PMC7307768; doi:10.1371/journal.pone.0234638)
Supplement: S2 Fig — A-B: Human HSCs were cultured in HSC medium supplemented with 1 mM VPA on uncovered 3D PDMS scaffolds for 14 DIV. All cell nuclei were stained with Draq5 (red) and dead cells were stained by DAPI uptake (blue) shortly before fixation. Z-stack images of whole scaffolds were taken on an Axio Scan.Z1 Slide Scanner microscope in Z-stacks before (A) and after (B) harvesting. The pictures show reconstructions of extended focus images 2D projection of multiple Z-stacks and are representative for three independent experiments. C-G: The absolute number, percentage and the viability of CD34+ cells was determined by flow cytometric analyses using a combination of anti-CD34/anti-CD45 mouse monoclonal antibody and 7AAD at days 0 and 14 of in vitro culture. (C) The percentage and absolute cell number of CD34+ cells after 0 DIV are depicted for four different donors as their mean ± SD. (D) The percentage, the absolute number and viability of CD34+ cells after 0 DIV are depicted for three different donors as their mean ± SD. (E) Human HSCs were cultured in HSC medium supplemented without or with 0.5 or 1 mM VPA on uncovered 3D PDMS scaffolds for 14 DIV. The fold amplification of all cells and CD34+ cells, the percentage and the absolute cell number of CD34+ cells are depicted for three different donors as their mean ± SD. (F) The corresponding viability of CD34+ cells after 14 DIV in the presence of 0.5 or 1 mM VPA is depicted for three different donors as their mean ± SD. (G) The percentage and absolute number of CD34+ cells after 0 DIV are depicted for nine different donors as their mean ± SD. (PPTX) [file pone.0234638.s002.pptx]

## Slide 1
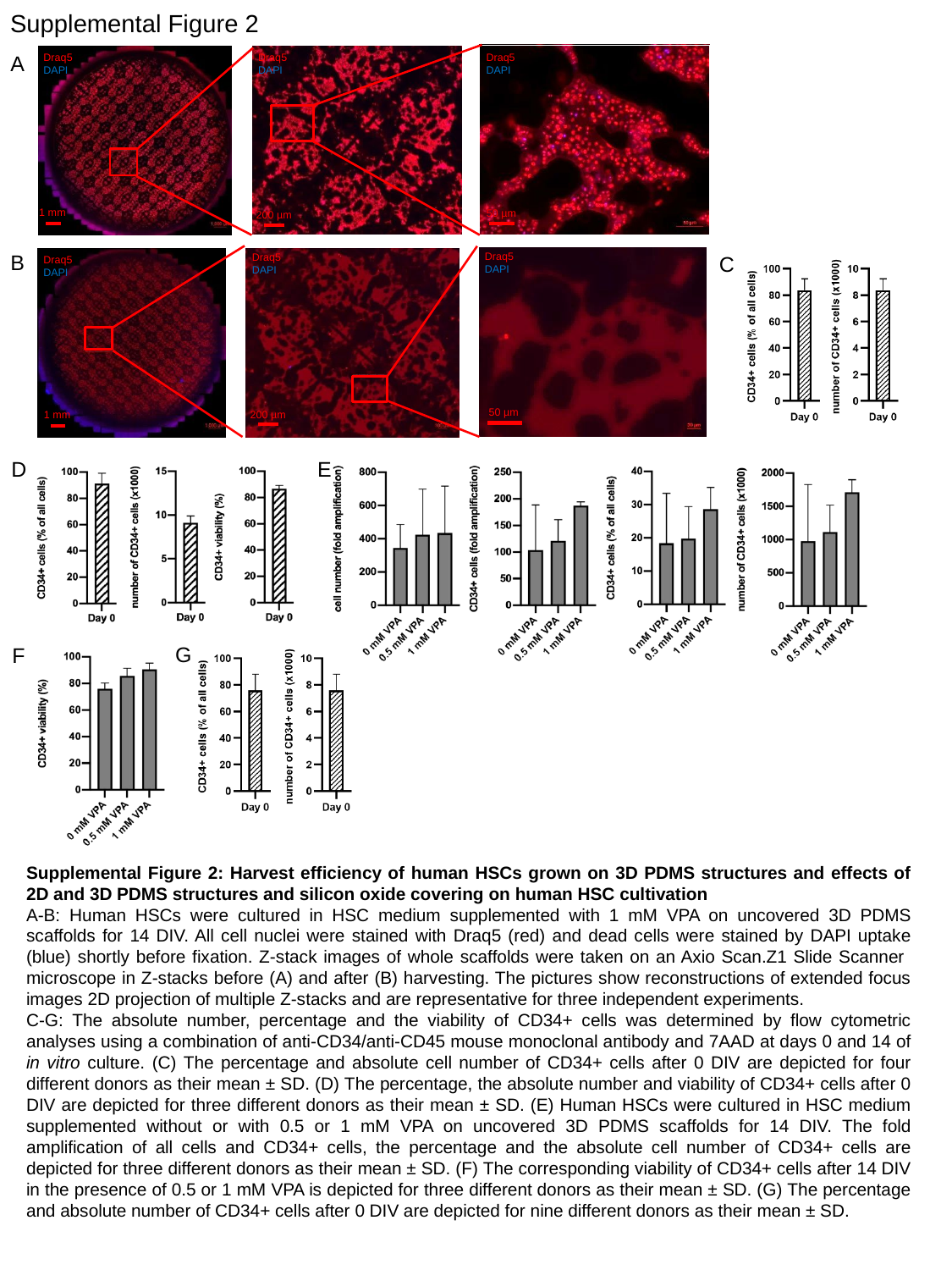

Supplemental Figure 2
A
Draq5
DAPI
Draq5
DAPI
Draq5
DAPI
1 mm
50 µm
200 µm
B
Draq5
DAPI
Draq5
DAPI
Draq5
DAPI
50 µm
1 mm
200 µm
C
D
E
G
F
Supplemental Figure 2: Harvest efficiency of human HSCs grown on 3D PDMS structures and effects of 2D and 3D PDMS structures and silicon oxide covering on human HSC cultivation
A-B: Human HSCs were cultured in HSC medium supplemented with 1 mM VPA on uncovered 3D PDMS scaffolds for 14 DIV. All cell nuclei were stained with Draq5 (red) and dead cells were stained by DAPI uptake (blue) shortly before fixation. Z-stack images of whole scaffolds were taken on an Axio Scan.Z1 Slide Scanner microscope in Z-stacks before (A) and after (B) harvesting. The pictures show reconstructions of extended focus images 2D projection of multiple Z-stacks and are representative for three independent experiments.
C-G: The absolute number, percentage and the viability of CD34+ cells was determined by flow cytometric analyses using a combination of anti-CD34/anti-CD45 mouse monoclonal antibody and 7AAD at days 0 and 14 of in vitro culture. (C) The percentage and absolute cell number of CD34+ cells after 0 DIV are depicted for four different donors as their mean ± SD. (D) The percentage, the absolute number and viability of CD34+ cells after 0 DIV are depicted for three different donors as their mean ± SD. (E) Human HSCs were cultured in HSC medium supplemented without or with 0.5 or 1 mM VPA on uncovered 3D PDMS scaffolds for 14 DIV. The fold amplification of all cells and CD34+ cells, the percentage and the absolute cell number of CD34+ cells are depicted for three different donors as their mean ± SD. (F) The corresponding viability of CD34+ cells after 14 DIV in the presence of 0.5 or 1 mM VPA is depicted for three different donors as their mean ± SD. (G) The percentage and absolute number of CD34+ cells after 0 DIV are depicted for nine different donors as their mean ± SD.
