# Supplementary material for: Biomimetic reconstruction of the hematopoietic stem cell niche for in vitro amplification of human hematopoietic stem cells
Source: PLoS One. 2020 Jun 22;15(6):e0234638. doi: 10.1371/journal.pone.0234638 (PMC7307768; doi:10.1371/journal.pone.0234638)
Supplement: S3 Fig — Human HSCs were cultured in HSC medium supplemented with 1 mM VPA on SiOn-covered and uncovered 3D PDMS scaffolds. All cell nuclei were stained with Draq5 (red) and dead cells were stained by DAPI uptake (blue) shortly before fixation. CD34+ cells were immune-stained after fixation with a primary anti-CD34 antibody in combination with an Alexa Fluor555-conjugated secondary antibody (green). Z-stack images of whole scaffolds were taken on an Axio Scan.Z1 Slide Scanner microscope in Z-stacks after 7 (A) and 14 (B) DIV. The pictures show reconstructions of extended focus images 2D projection of multiple Z-stacks and are representative for three independent experiments. Detail images of selected areas were taken with an Axio Imager equipped with an ApoTome.2 slider ApoTome microscope for optical sectioning in Z-stacks after 7 and 14 DIV (C). The pictures show orthogonal 2D projection of multiple Z-stacks and are representative for three independent experiments. (PPTX) [file pone.0234638.s003.pptx]

## Slide 1
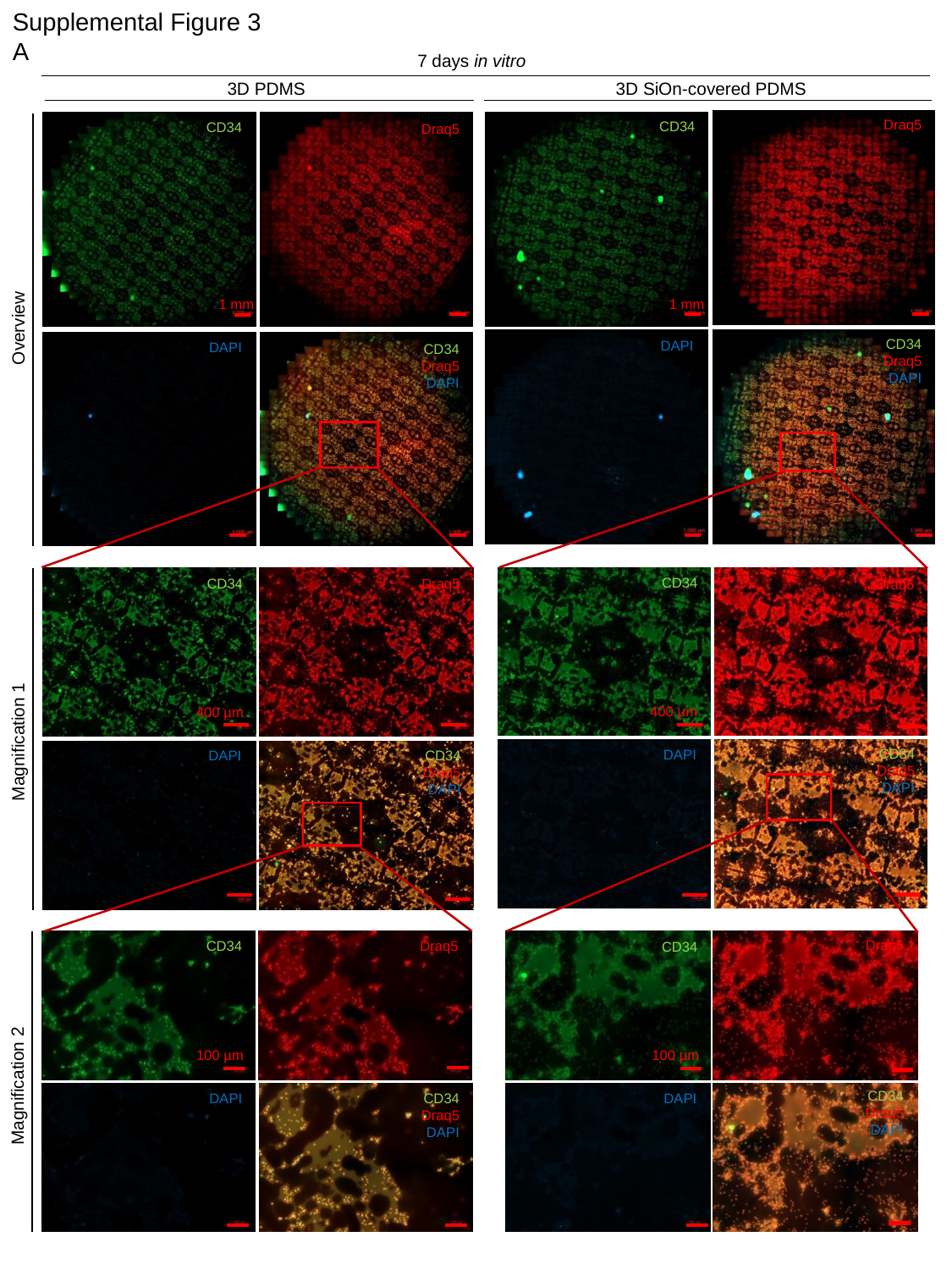

3D SiOn-covered PDMS
3D PDMS
Supplemental Figure 3
A
7 days in vitro
Draq5
CD34
CD34
Draq5
CD34
Draq5
DAPI
DAPI
DAPI
CD34
Draq5
DAPI
CD34
Draq5
CD34
Draq5
CD34
Draq5
DAPI
DAPI
DAPI
CD34
Draq5
DAPI
Draq5
CD34
Draq5
CD34
CD34
Draq5
DAPI
DAPI
DAPI
CD34
Draq5
DAPI
Overview
Magnification 1
Magnification 2
1 mm
1 mm
400 µm
400 µm
100 µm
100 µm

## Slide 2
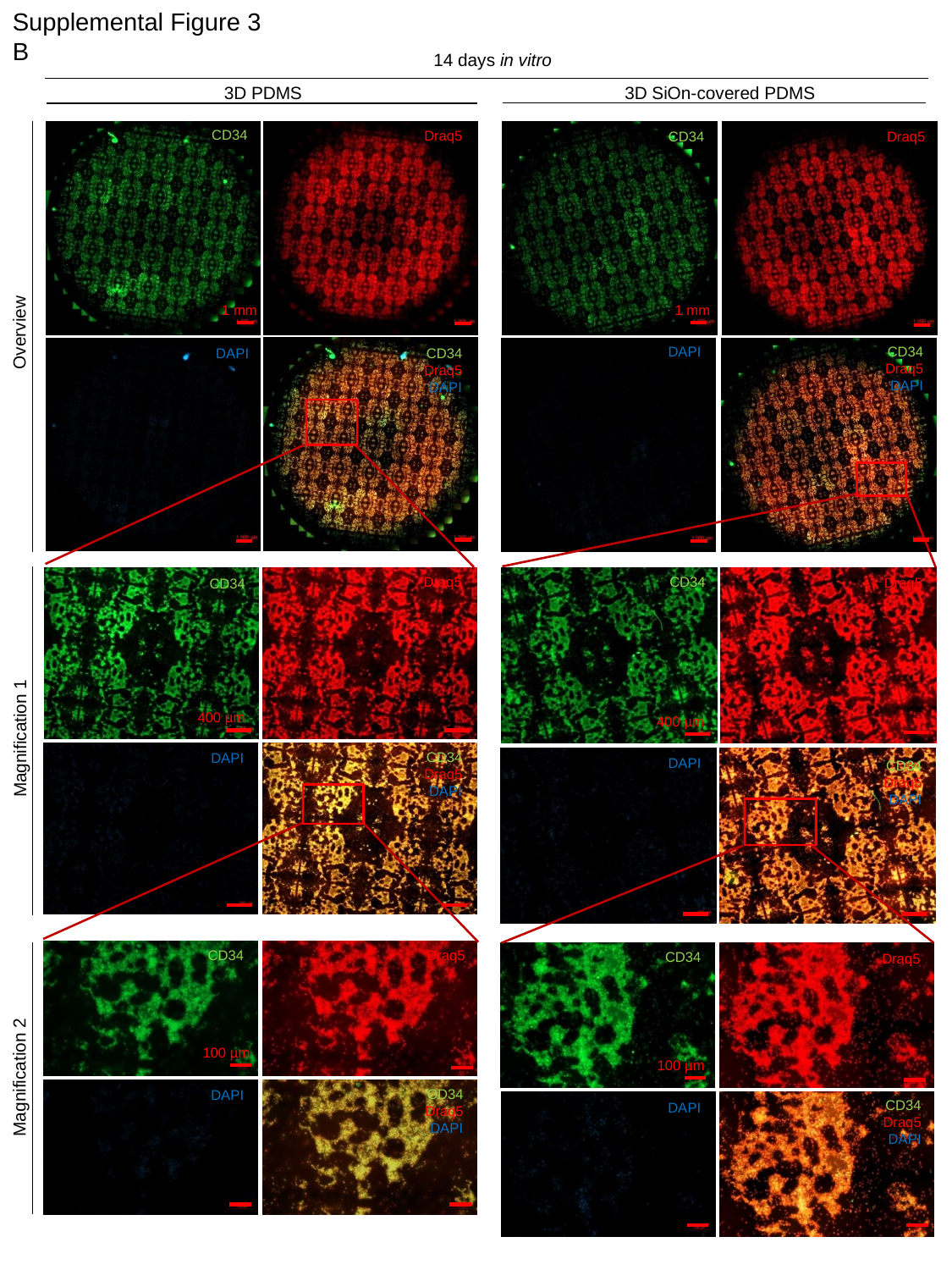

Supplemental Figure 3
B
3D SiOn-covered PDMS
3D PDMS
14 days in vitro
CD34
Draq5
Draq5
CD34
DAPI
CD34
Draq5
DAPI
DAPI
CD34
Draq5
DAPI
Draq5
CD34
Draq5
CD34
CD34
Draq5
DAPI
DAPI
DAPI
CD34
Draq5
DAPI
CD34
Draq5
CD34
Draq5
CD34
Draq5
DAPI
DAPI
CD34
Draq5
DAPI
DAPI
Overview
Magnification 1
Magnification 2
1 mm
1 mm
400 µm
400 µm
100 µm
100 µm

## Slide 3
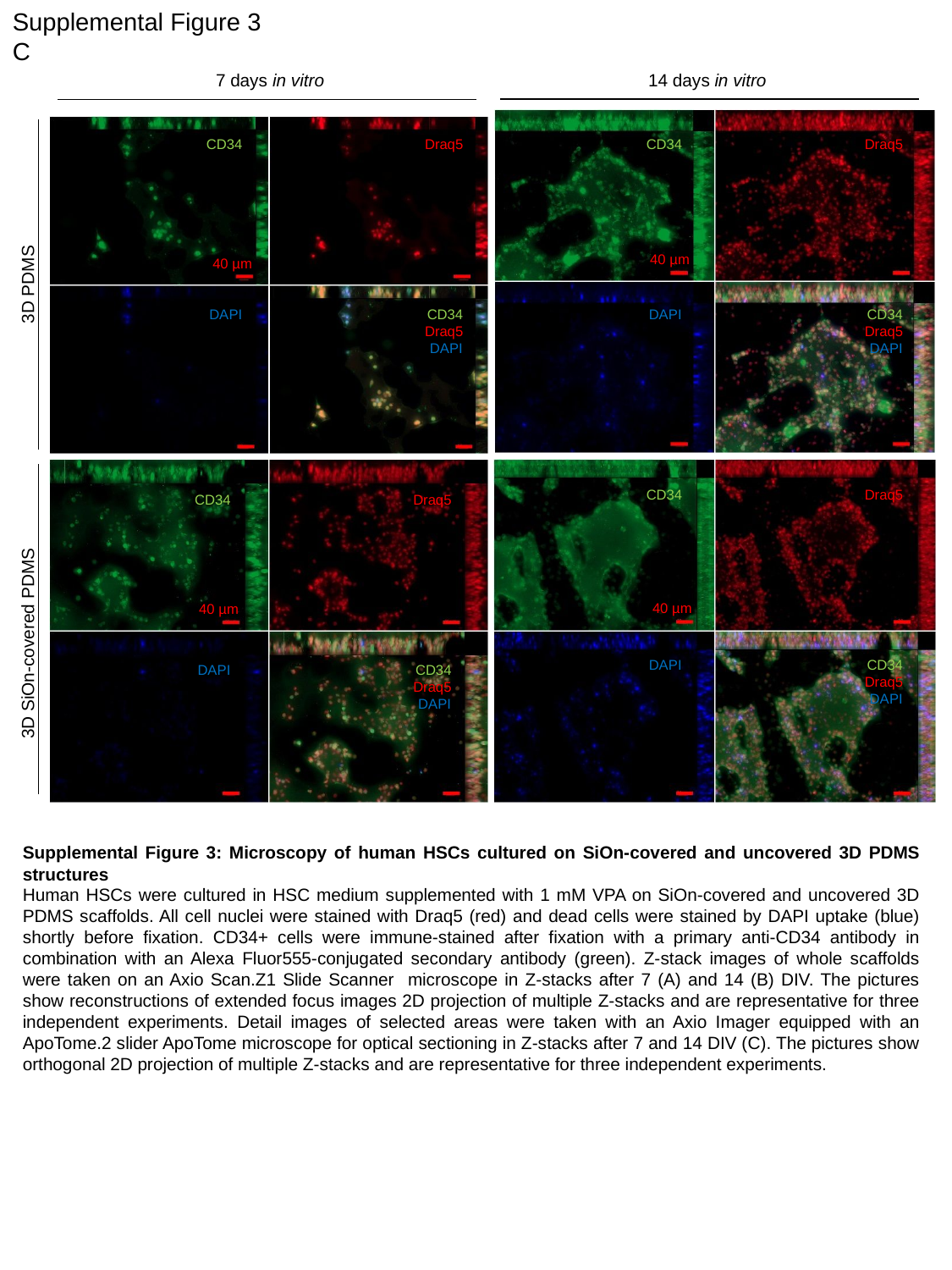

Supplemental Figure 3
C
7 days in vitro
14 days in vitro
CD34
Draq5
CD34
Draq5
40 µm
40 µm
3D PDMS
DAPI
CD34
Draq5
DAPI
DAPI
CD34
Draq5
DAPI
CD34
Draq5
CD34
Draq5
40 µm
40 µm
3D SiOn-covered PDMS
DAPI
CD34
Draq5
DAPI
DAPI
CD34
Draq5
DAPI
Supplemental Figure 3: Microscopy of human HSCs cultured on SiOn-covered and uncovered 3D PDMS structures
Human HSCs were cultured in HSC medium supplemented with 1 mM VPA on SiOn-covered and uncovered 3D PDMS scaffolds. All cell nuclei were stained with Draq5 (red) and dead cells were stained by DAPI uptake (blue) shortly before fixation. CD34+ cells were immune-stained after fixation with a primary anti-CD34 antibody in combination with an Alexa Fluor555-conjugated secondary antibody (green). Z-stack images of whole scaffolds were taken on an Axio Scan.Z1 Slide Scanner microscope in Z-stacks after 7 (A) and 14 (B) DIV. The pictures show reconstructions of extended focus images 2D projection of multiple Z-stacks and are representative for three independent experiments. Detail images of selected areas were taken with an Axio Imager equipped with an ApoTome.2 slider ApoTome microscope for optical sectioning in Z-stacks after 7 and 14 DIV (C). The pictures show orthogonal 2D projection of multiple Z-stacks and are representative for three independent experiments.
