# Supplementary material for: Biomimetic reconstruction of the hematopoietic stem cell niche for in vitro amplification of human hematopoietic stem cells
Source: PLoS One. 2020 Jun 22;15(6):e0234638. doi: 10.1371/journal.pone.0234638 (PMC7307768; doi:10.1371/journal.pone.0234638)
Supplement: S4 Fig — Cells were stained with DAPI and monoclonal antibodies conjugated with different fluorophores against CD34, CD38, CD49f, CD45RA and CD90. Prior to flow cytometric analyses of CD34+ and CD34+/ CD38-/ CD45RA-/ CD49f+/ CD90+ cells, a doublet discrimination was done and vital cells were selected using DAPI. According to the IgG control to every antibody (not shown), gates were set and distinct populations were determined. (PPTX) [file pone.0234638.s004.pptx]

## Slide 1
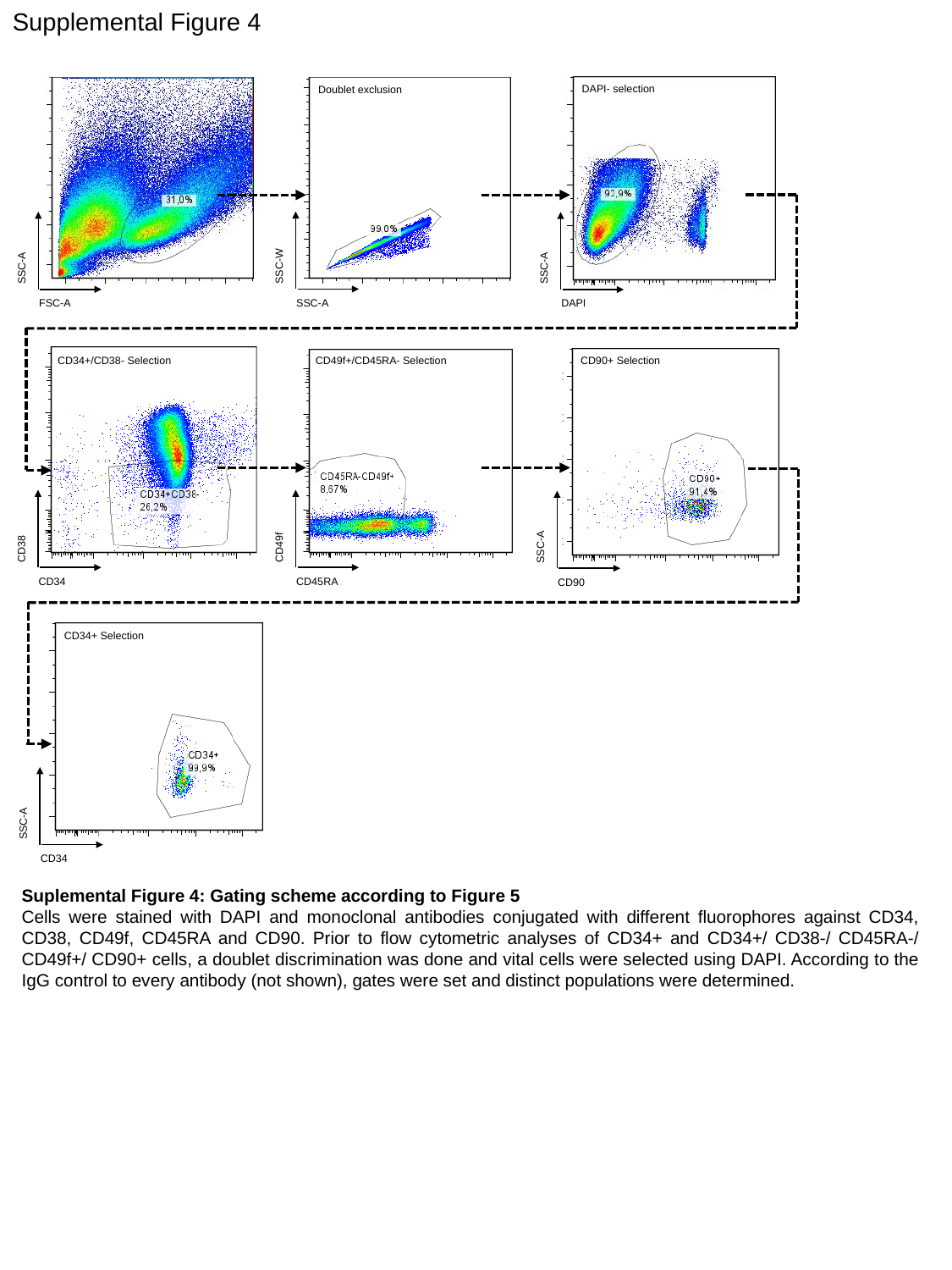

Supplemental Figure 4
DAPI- selection
Doublet exclusion
SSC-W
SSC-A
SSC-A
SSC-A
FSC-A
DAPI
CD34+/CD38- Selection
CD49f+/CD45RA- Selection
CD90+ Selection
CD38
CD49f
SSC-A
CD34
CD45RA
CD90
CD34+ Selection
SSC-A
CD34
Suplemental Figure 4: Gating scheme according to Figure 5
Cells were stained with DAPI and monoclonal antibodies conjugated with different fluorophores against CD34, CD38, CD49f, CD45RA and CD90. Prior to flow cytometric analyses of CD34+ and CD34+/ CD38-/ CD45RA-/ CD49f+/ CD90+ cells, a doublet discrimination was done and vital cells were selected using DAPI. According to the IgG control to every antibody (not shown), gates were set and distinct populations were determined.
