# Supplementary material for: Biomimetic reconstruction of the hematopoietic stem cell niche for in vitro amplification of human hematopoietic stem cells
Source: PLoS One. 2020 Jun 22;15(6):e0234638. doi: 10.1371/journal.pone.0234638 (PMC7307768; doi:10.1371/journal.pone.0234638)
Supplement: S5 Fig — CD34+ and CD34+/ CD38-/ CD45RA-/ CD49f+/ CD90+ HSC cells after 0 DIV were determined by flow cytometric analyses using a combination of specific antibodies and vital cells were selected using DAPI. The percentage and absolute number of CD34+ cells as well as the percentage and the absolute number of CD34+/ CD38-/ CD45RA-/ CD49f+/ CD90+ cells are depicted for three different donors as their mean ± SD (A and B show independent experiments). (PPTX) [file pone.0234638.s005.pptx]

## Slide 1
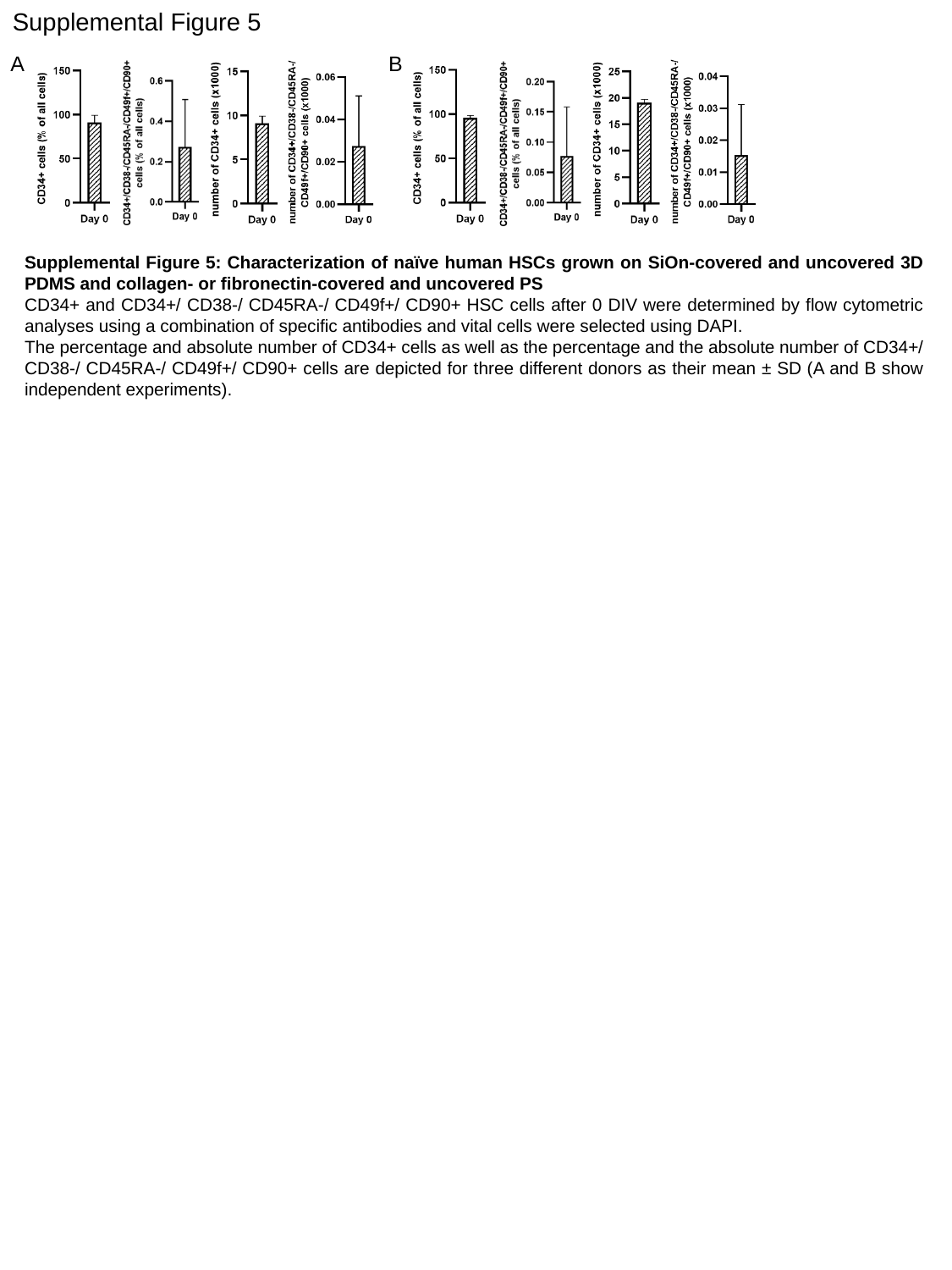

Supplemental Figure 5
B
A
Supplemental Figure 5: Characterization of naïve human HSCs grown on SiOn-covered and uncovered 3D PDMS and collagen- or fibronectin-covered and uncovered PS
CD34+ and CD34+/ CD38-/ CD45RA-/ CD49f+/ CD90+ HSC cells after 0 DIV were determined by flow cytometric analyses using a combination of specific antibodies and vital cells were selected using DAPI.
The percentage and absolute number of CD34+ cells as well as the percentage and the absolute number of CD34+/ CD38-/ CD45RA-/ CD49f+/ CD90+ cells are depicted for three different donors as their mean ± SD (A and B show independent experiments).
