# Supplementary material for: Biomimetic reconstruction of the hematopoietic stem cell niche for in vitro amplification of human hematopoietic stem cells
Source: PLoS One. 2020 Jun 22;15(6):e0234638. doi: 10.1371/journal.pone.0234638 (PMC7307768; doi:10.1371/journal.pone.0234638)
Supplement: S6 Fig — A: Burst-forming unit-erythroid (BFU-E), B: Colony-forming unit-granulocyte/erythroid/megakaryocyte/monocyte (CFU-GEMM), C: Colony-forming unit-erythroid (CFU-E), D: Colony-forming unit-macrophage (CFU-M), E: Colony-forming unit-granulocyte (CFU-G), F: Colony-forming unit-granulocyte/macrophage (CFU-GM). Images were examined using a Zeiss AxioVert 40 CFL microscope and an AxioCam MRc 5 (Carl Zeiss) after 14 days of incubation in multi-lineage CFU medium. (PPTX) [file pone.0234638.s006.pptx]

## Slide 1
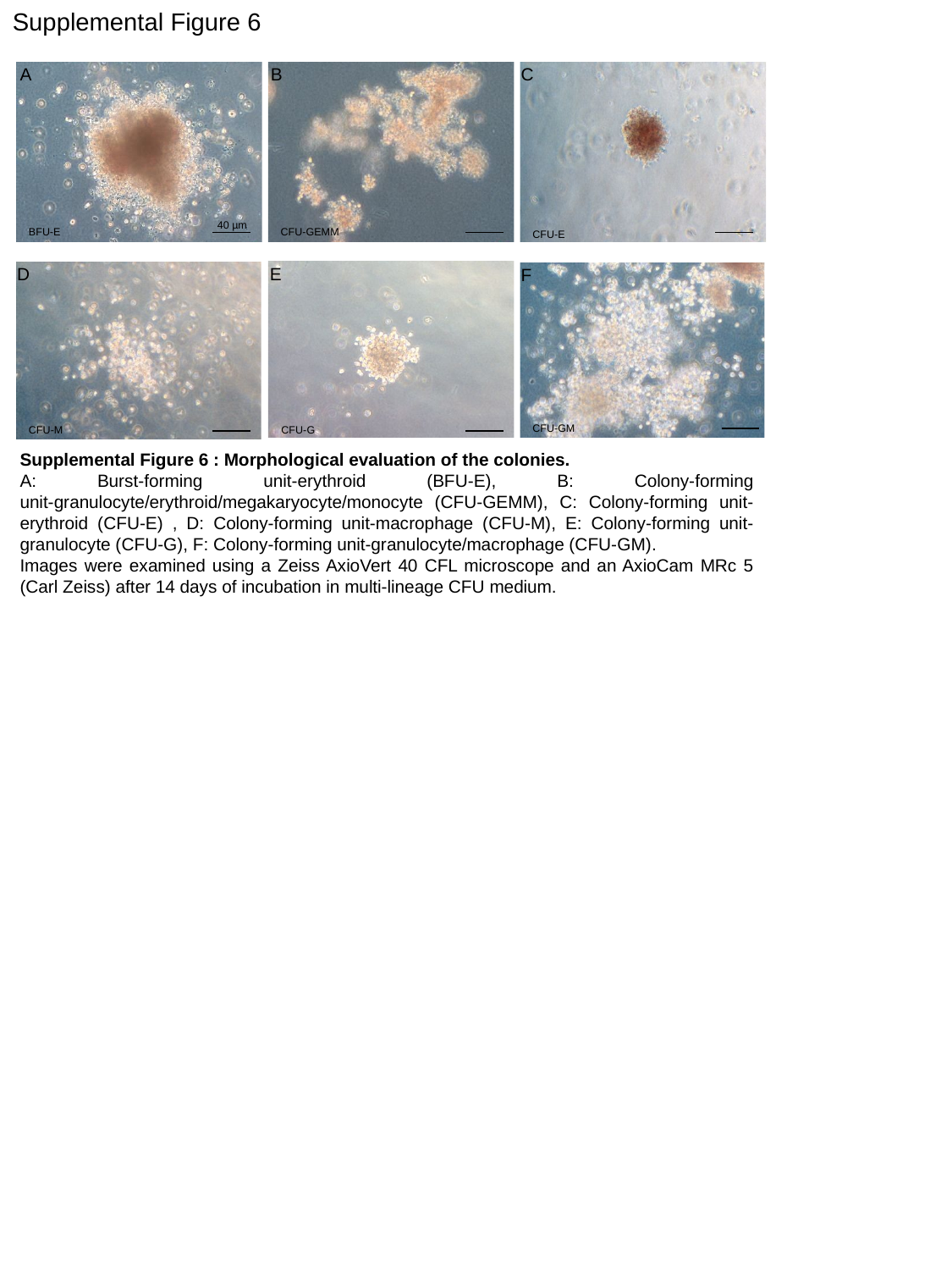

Supplemental Figure 6
A
B
C
BFU-E
CFU-GEMM
CFU-E
CFU-GM
CFU-M
CFU-G
40 µm
D
E
F
Supplemental Figure 6 : Morphological evaluation of the colonies.
A: Burst-forming unit-erythroid (BFU-E), B: Colony-forming unit-granulocyte/erythroid/megakaryocyte/monocyte (CFU-GEMM), C: Colony-forming unit-erythroid (CFU-E) , D: Colony-forming unit-macrophage (CFU-M), E: Colony-forming unit-granulocyte (CFU-G), F: Colony-forming unit-granulocyte/macrophage (CFU-GM).
Images were examined using a Zeiss AxioVert 40 CFL microscope and an AxioCam MRc 5 (Carl Zeiss) after 14 days of incubation in multi-lineage CFU medium.
